# Finding the forgotten gems: revisiting the butterflies of Matheran after 125 years with introduction to novel colour barcode for depicting seasons and activity of the Indian butterflies

**DOI:** 10.3897/BDJ.8.e54333

**Published:** 2020-08-07

**Authors:** Mandar Sawant, Sagar Sarang, Nikhil Modak

**Affiliations:** 1 Bombay Natural History Society, Hornbill House, Shahid Bhagat Singh Rd, Lion Gate, Fort, Mumbai, Maharashtra 400001, Mumbai, India Bombay Natural History Society, Hornbill House, Shahid Bhagat Singh Rd, Lion Gate, Fort, Mumbai, Maharashtra 400001 Mumbai India; 2 Department of Zoology, Somaiya Vidya Vihar University, Vidya Vihar (East), 400077, Mumbai, India Department of Zoology, Somaiya Vidya Vihar University, Vidya Vihar (East), 400077 Mumbai India; 3 106, Kalpataru Tower CHSL, N/r. Sakharam Complex, Kopar Cross Road, Dombivli (West), 421202, Dombivli (West), India 106, Kalpataru Tower CHSL, N/r. Sakharam Complex, Kopar Cross Road, Dombivli (West), 421202 Dombivli (West) India

**Keywords:** Lepidoptera, Eco-sensitive zone, biodiversity hotspot, colour barcode

## Abstract

We present here an updated checklist for the butterflies of Matheran, Maharashtra, India, an eco-sensitive zone, with identification remarks for locally rare or very rare butterflies. This is the first dedicated checklist for butterflies of Matheran after 125 years. A total of 140 species of butterflies were recorded belonging to six families. Amongst them, 15 species were either listed under Schedule I, II or IV of the Indian Wildlife (Protection) Act, 1972. We also list the habitats of the species along with the data for their activity at the time of recording the observation. We propose a uniform colour code system for representing season and activity for the Indian butterflies. Examples of colour barcodes are provided with the images of rare and very rare butterflies. The lack of abundance data is a limitation of the study for which we propose long term monitoring with dedicated efforts.

## Introduction

Butterflies are an ideal taxonomic group for ecological studies of landscapes (Thomas and Malorie 1985) and their value as indicators of biotope quality is being increasingly recognised because of their sensitivity to minor changes in micro-habitat, particularly to the luminosity (Kremen 1992). Further, the butterflies are good biological indicators of habitat quality, as well as for the general health of the environment (Larsen 1988; Kocher and Williams 2000; Sawchik et al. 2005). Long-term diversity studies could, therefore, indicate the health of the habitat and ecosystems therein.

Here, we provide a checklist for butterflies of Matheran surveyed between the years 2011 and 2019. Ours is the first dedicated checklist for the butterflies of Matheran after Betham (1894). He listed 78 species of butterflies, combining the list of sixty butterflies provided by Smith (1882) and the list of butterflies recorded by him between April and May 1892. Padhye et al. (2013) provided a list of 27 butterflies from Matheran, while compiling the checklists for the butterflies of Northern Western Ghats, which was far from complete when compared to that given by Betham (1894). Further, the data on the habitat and seasonal turnover for butterflies of Matheran are particularly lacking from all these studies. Our checklist is accompanied with data on habitat, seasonal turnover and behavioural observations taken at the time of recording the species. We provide a novel coloured barcode approach for indicating the season/s and types of behaviour which could be used for all Indian butterflies. Representative colour barcodes are provided with the images of rare and scheduled species.

## Materials and Methods

### Study Area

**Figure 1.**
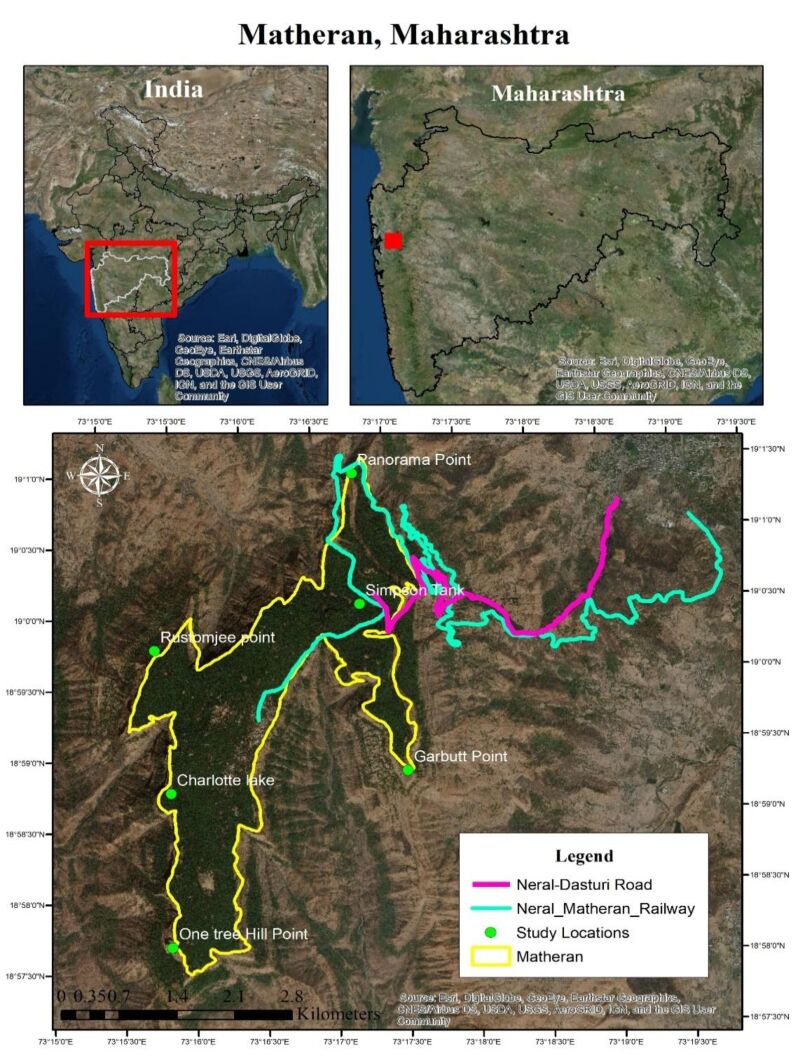
Study area with its location in Maharashtra, India. Sampling sites are shown in green filled circles. Additionally, the survey was conducted on two trails, Neral-Dasturi Road (pink line) and Neral-Matheran Railway (green line).

Matheran (18.9866°N 73.2679°E, 772 m a.s.l., WGS 84) is a small hill station located in Karjat Tehsil of Raigad District in the Indian State of Maharashtra (Fig. 1). It is spread over an area of 7 sq. km. Matheran literally means forest on the top of the mountains. Geologically, it is a basaltic mesa separated from the main escarpment of Western Ghats by the low lying plains of Konkan and is an example of regressive erosion (Pascal 1988). Matheran gained the status of an Eco-Sensitive Zone (ESZ) in 2003 from the Ministry of Environment, Forest and Climate Change, Government of India [S. O. 133 (E)]. The ESZ of the Matheran comprises an area of 214.73 sq. km. All types of industrial, developmental and vehicular activities are restricted by this governmental order, making Matheran unique amongst hill stations of Asia. It experiences a cooler climate throughout the year (23.2°C mean annual temperature) compared to the surrounding low lying area and experiences heavy rainfall during the monsoon (4073 mm mean annual rainfall). The landscapes of Matheran are represented by open or forested laterite plateaus, hill-slopes, dense valley forests, non-perennial streams, manmade lakes, clearings near forest paths and human habitation. The flora of Matheran is represented by tree species found in mid elevation type wet evergreen forest (Ramesh et al. 1997), dominated by *Memecylon umbellatum*, *Syzygium cumini* and *Actinodaphne lanceolata* (Birdwood 1886, Ramesh et al. 1997). The plateau also hosts species like *Carallia integerrima*, *Glochidion lanceolarium*, *Olea dioica*, *Garcinia indica* and *Carissa carandas* (Birdwood 1886). The area also shows the presence of many endemic species of orchids, grasses and other herbaceous plants (Kothari and Moorthy 1993).

### Field Survey and Data Collection

The area was visited in all the three seasons, namely summer (Feb-May), monsoon (Jun-Sept) and winter (Oct-Jan) throughout the year from September 2011 to March 2019. Intermittent observations were taken between 06.00 hrs and 17.00 hrs for around three days a month. The butterflies were observed in all possible habitats at six localities and on two trails in and around Matheran (Table 1). A total of 22833 observations were made during nine years of the study (https://indiabiodiversity.org/dataTable/show/1755286) which are available as a data table on the India Biodiversity Portal (Vattakaven et al. 2016). To ascertain the identity of butterflies, photographs were taken and identifications were made with the keys provided by Evans (1932), Wynter-Blyth (1957), Kunte (2000), Kehimkar (2008), Kehimkar (2016) and Bhakare and Ogale (2018). The classification and nomenclature follows Kehimkar (2008), Van Gasse (2013) and Varshney and Smetacek (2015). The local status of the butterflies was decided, based on the number of records as very rare (≤ 5 records), rare (between 5 and 10), not common (between 10 and 20), common (between 20 and 50) and very common (> 50). This status does not correlate to the entire geographical distribution status of a corresponding species. The habitat, occurrence and behaviour of butterflies were noted and photo documented. The photo documentation was made with Nikon d500, d3200 and Cannon EOS 70d, Sony HX 100v digital cameras. The species were noted along with the date and location.

### Data Analysis

Based on the occurrence data, a species accumulation curve (SAC) was prepared in R (R Core Team 2020) using the SpecAccum function in vegan (Oksanen et al. 2019). Expected (mean) species richness was calculated using the data collected from eight sites (Table 1). Further, the occurrence data of the species were analysed for calculating Similarity-Richness difference-Species replacement simplex (SDR Simplex) using SDRSimplex (a stand-alone computer programme) (Podani and Schmera 2011). Ternary plots were plotted using NonHier platform of SYNTAX 2000 (Podani 2001). The number or percentage of the species recorded per family, during each season, at each site was calculated in Microsoft Excel 2007 and visualised using pie and bar charts.

### Preparation of Colour codes

The colour codes (Table 2) were prepared for easy and uniform representation of seasons and various behavioural activities of the Indian butterflies. Summer, monsoon and winter were given basic red, green and indigo colours in the CMYK scheme. These colours also correspond to temperature shifts in the seasons from hotter to cooler weather conditions. For combination of seasons, the corresponding combination of colours was used. Colours were mixed online through Color Mixer platform of Color Designer (https://colordesigner.io/color-mixer). Grey colour represents the occurrence of the species in all seasons. All other colours were selected from the RGB scheme for it provides a wider range of colours. These colours were selected in such a way that they represent the correponding activity, for example, brown for mud puddling, honey colour (orange palette) for nectaring, amber colour for tree sap feeding etc., except basking which is represented by magenta.

## Results

### Species Richness

**Figure 2.**
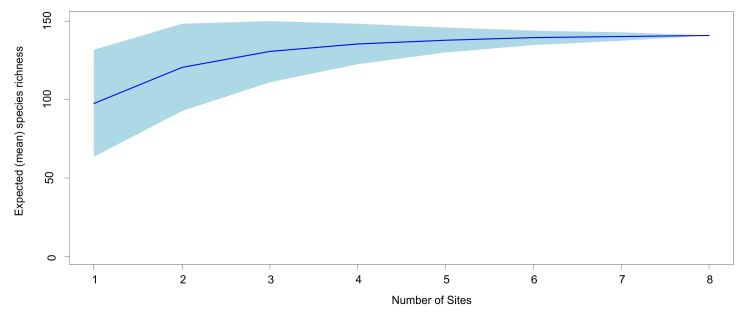
Species Accumulation Curve (SAC) with asymptote model. Dark blue line indicates the expected (mean) species richness; shaded area denotes the standard deviation (Asym = 146.42, xmid = 0.58, slope = 3.60).

**Figure 3.**
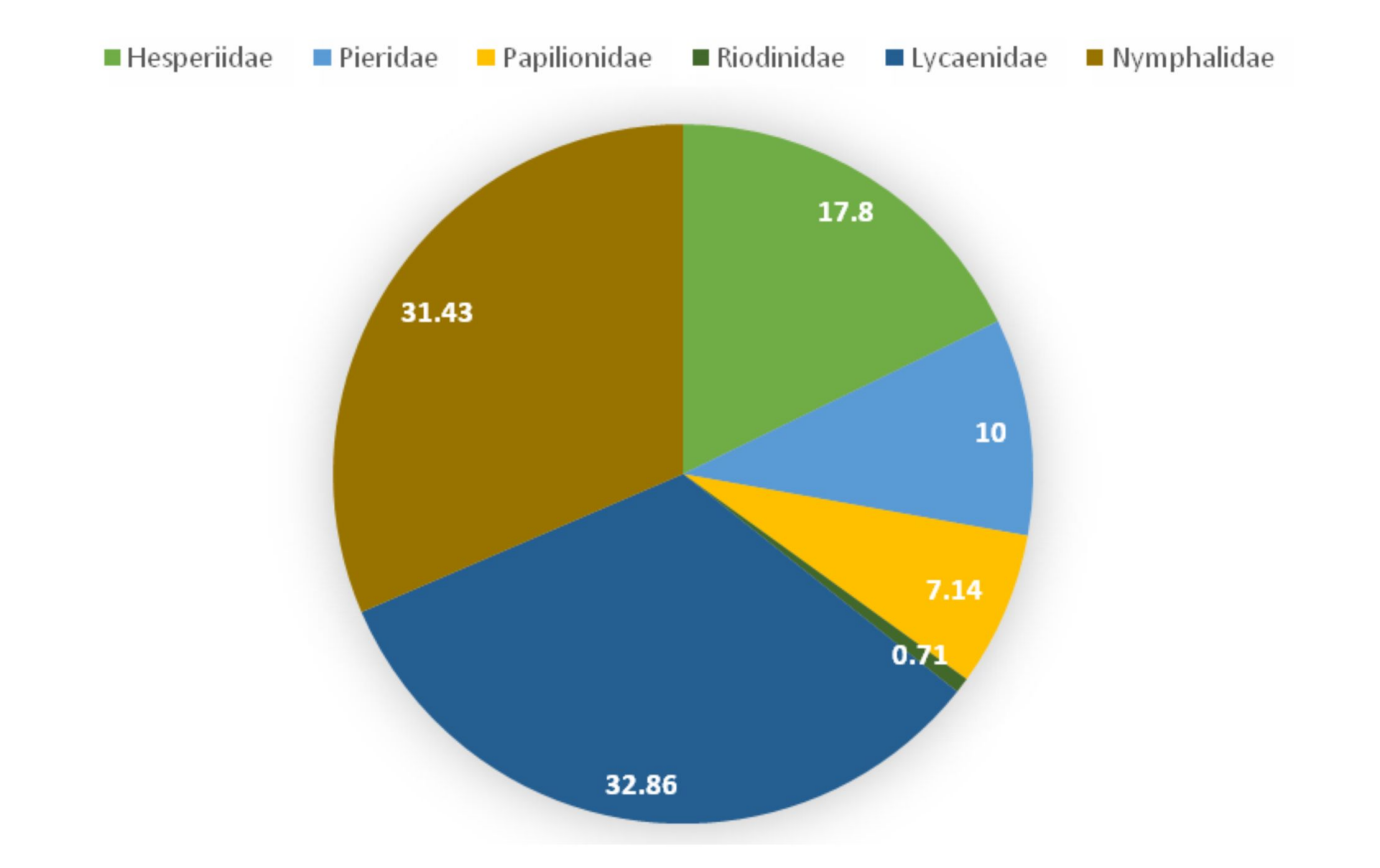
Family-wise species composition pie of butterflies of Matheran.

The SAC gained a plateau and standard deviation for species richness declined from 97.75 ± 17.07 to 141.0 ± 0.0 as the number of sights increased from one to eight, predicting sufficient efforts to record all the species found in the area (Asym = 146.42, xmid = 0.58, slope = 3.60) (Fig. 2). A total of 140 species belonging to six families have been observed and identified during the entire period of the study (Fig. 3, Table 3). The family Lycaenidae with 46 species (32.86%), followed by Nymphalidae with 43 species (31.43%), were amongst the most species-rich families in the area. Species belonging to the family Hesperiidae (25 species), Pieridae (14 species) and Papilionidae (10 species) were amongst other common species found in the area. The range of *Cheritra freja* (Common Imperial) which was earlier recorded from Amboli, Sindhudurga, Maharashtra (15.9647°N, 74.0036°E) (Saji and Ogale 2020) is extended further north around 345 km linear distance (calculated on https://www.nhc.noaa.gov/gccalc.shtml). The family Riodinidae was represented by only one species namely, *Abisara bifasciata* (Double Banded Judy).

### Seasonal turnover

**Figure 4.**
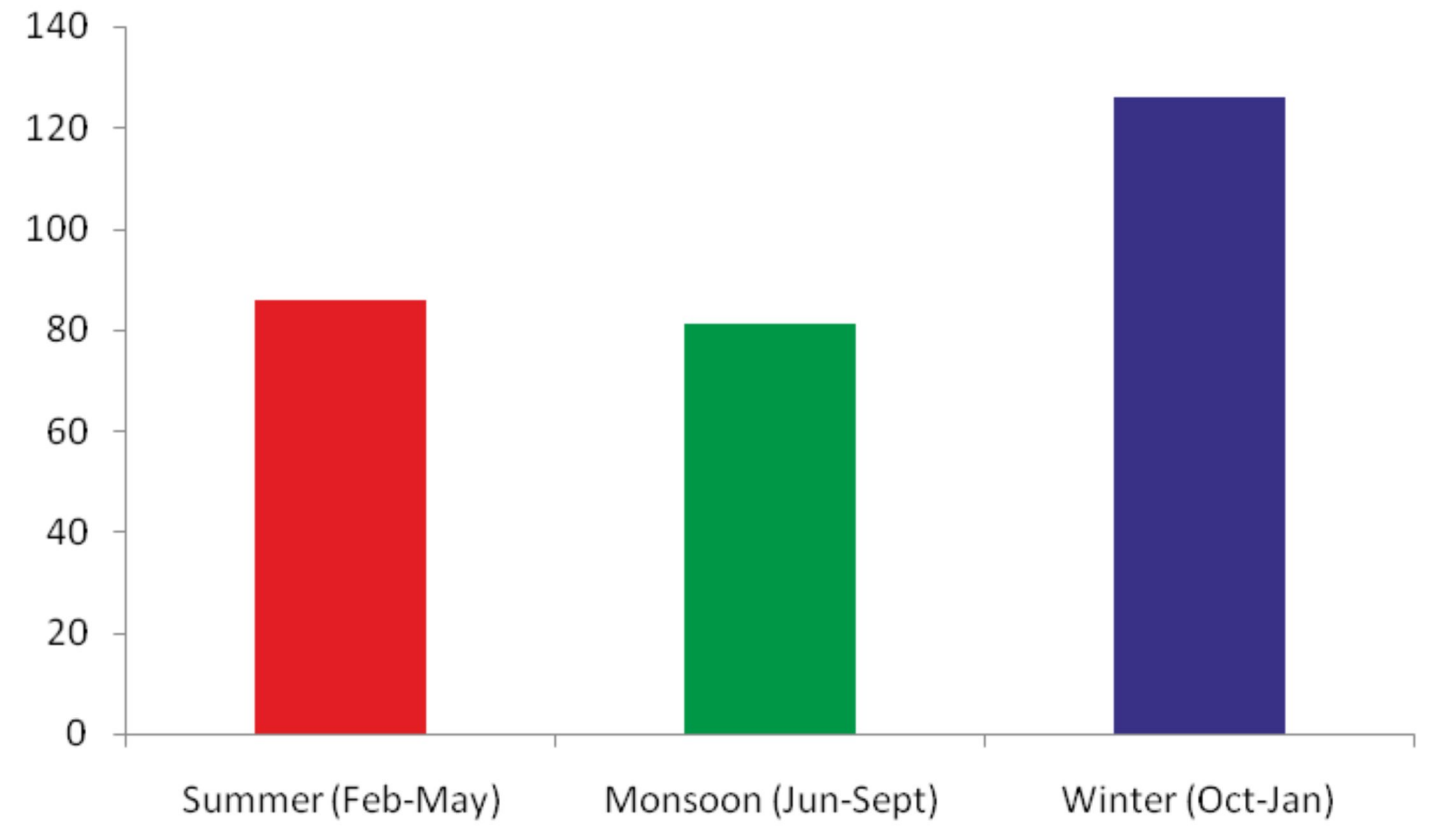
Seasonal variations in species richness.

**Figure 5.**
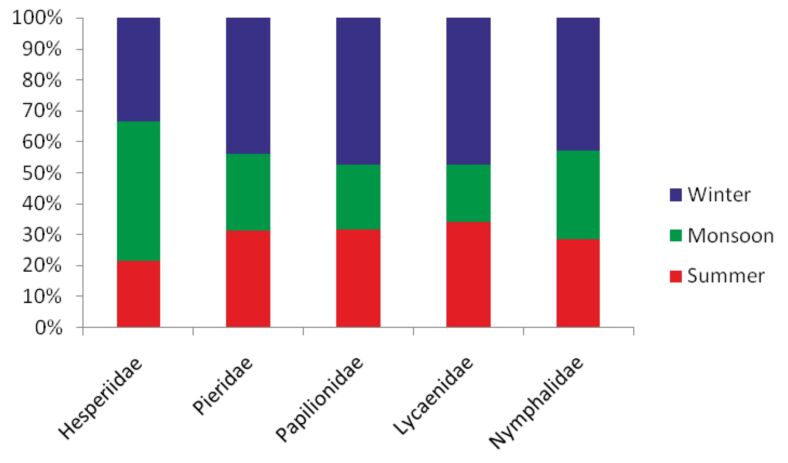
Family-wise percent species richness per season.

**Figure 6.**
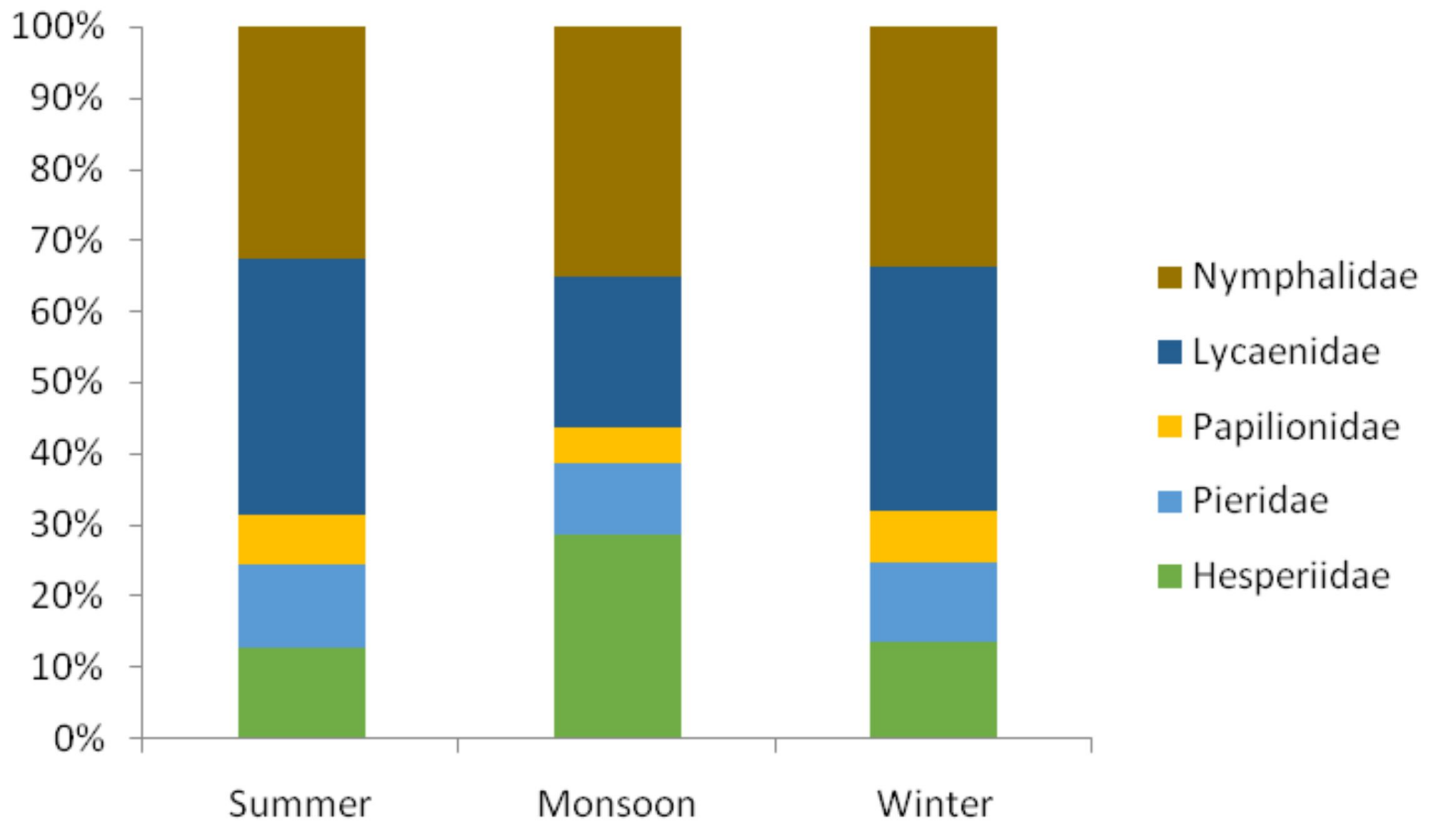
Season-wise percent species richness per family.

The maximum numbers of species (N = 125) were recorded during winter, while minimum numbers of species (N = 80) were recorded during the monsoon (Fig. 4). Maximum numbers of species for all the families were recorded during winter, except the family Hesperiidae for which the maximum numbers of species (N = 23) were recorded during the monsoon (Fig. 5). The species of the family Lycaenidae dominated the local butterfly species richness during the months of summer and winter with 36.05% (N = 31) and 34.40% (N = 43) of total species of butterflies recorded during respective seasons (Fig. 6). Members of the family Nymphalidae shared fairly equal percentages during all seasons. The percentage of the papilionids was the lowest during all seasons.

### Spatial turnover

**Figure 7.**
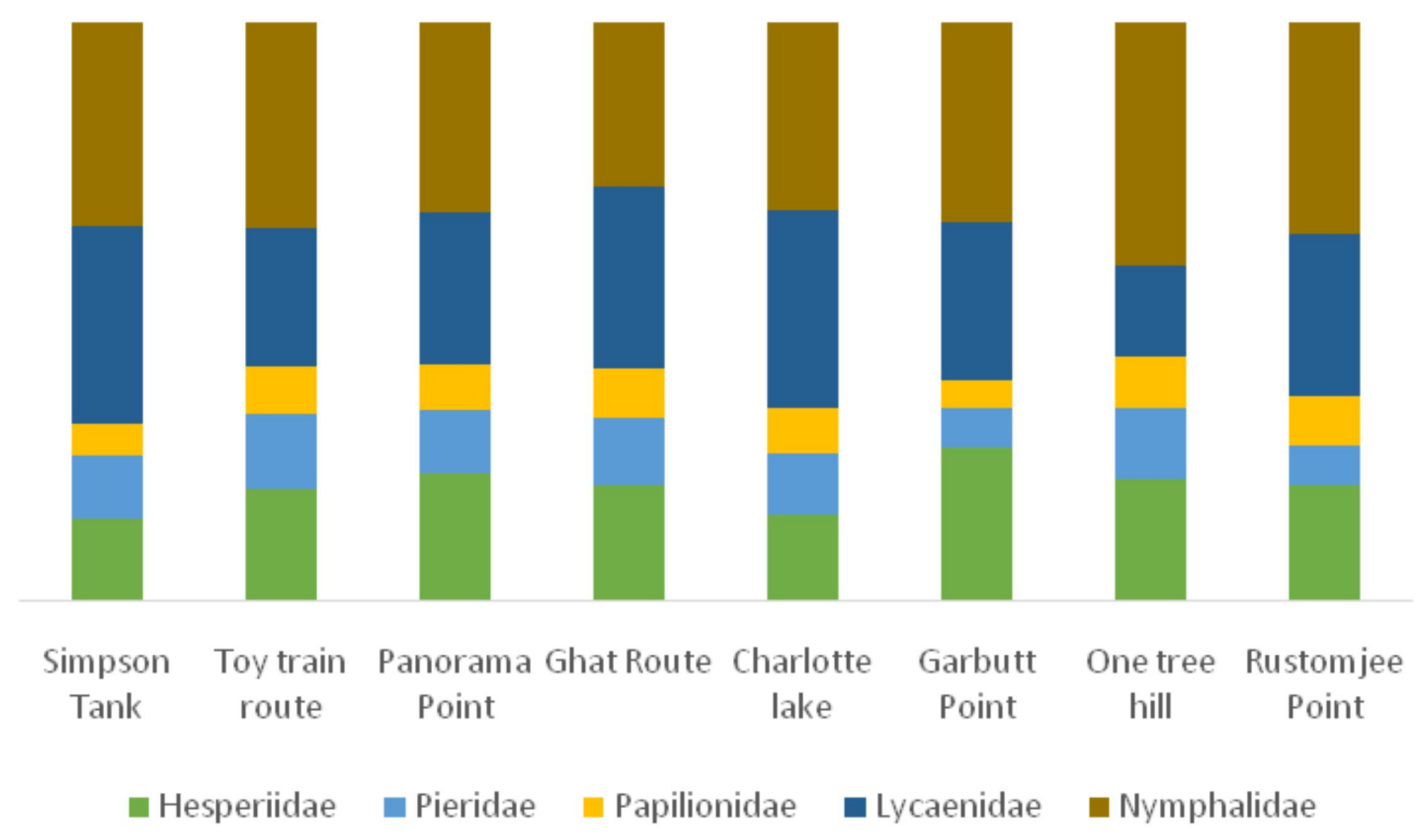
Site-wise percent species richness for each family

**Figure 8.**
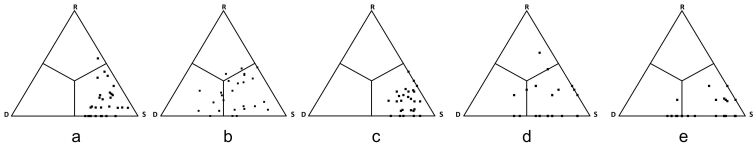
Similarity-Richness difference-Species replacement simplex plot for a. Hesperiidae; b. Lycaenidae; c. Nymphalidae; d. Papilionidae; e. Pieridae. S - Species Shared (Similarity); D - Richness difference; R - Species replacement. Squares indicate true simplex scores for each pairs of sites (N = 28 for 8 sites).

Members of the family Nymphalidae and Lycaenidae dominated the species diversity at all the sites studied in and around Matheran. Members of the family Lycaenidae were particularly present in higher numbers at Charlotte Lake while those of Hesperiidae were particularly present in higher numbers at Garbett Point (Fig. 7). The Similarity-Richness difference-Species replacement simplex for all the families indicated high similarity, although with different patterns tending towards perfect nestedness (Fig. 8a-e, Suppl. material 1). Similarity was the highest for the family Nymphalidae (70.58%) with 78.22% of relativised strict nestedness (nestedness without considering the effect of species replacement) and lowest relativised beta diversity of 29.42%. Relativised strict nestedness was the highest (85.67%) for the family Hesperiidae with a similarity of 65.91% and beta diversity of 34.10%, while relativised nestedness (nestedness considering the effect of species replacement) was the highest (93.56%) for the family Pieridae. Similarity of species composition between the sites was the lowest (49.10%) for the family Lycaenidae with the highest relativised richness difference (31.99%) indicating more site specific species composition for the members of the family Lycaenidae, unlike the members of other families.

### Activity of butterflies

No seasonal activity pattern could be observed (Table 3, Table 4). Most of the species were observed while mud puddling, basking or feeding on the nectar. Other common activities included feeding on bird droppings, tree sap, animal waste (other than that of birds) and/or animal carcasses.

### Locally rare and scheduled species

Our list contains 15 such species which are scheduled under the Wildlife (Protection) Act, 1972 of India (Table 5). Out of these, seven species were found rarely during the survey. Additionally, 20 species, which are not scheduled under the act, were observed rarely or very rarely during the survey (Figs 9, 10, 11, 12, 13)

### Identification remarks for locally rare or very rare butterflies

Abbreviations: FW-Forewing, HW-Hindwing, UN-Underside, UNF-Underside of Forewing, UNH-Underside of Hindwing, UP- Upperside, UPF-Upperside of Forewing, UPH-Upperside of Hindwing

#### Family Hesperiidae Latreille, 1809

**Genus *Bibasis* Moore, 1881**

*Bibasis sena* (Moore, 1865) (Fig. 9a).

Common name: Orange-tailed awlet.

Identification remarks: Bright orange fringe on HW and on the tip of the abdomen. Broad, pure white, outwardly diffused, central band on UN. Wingspan 42–50 mm.

Season: Monsoon.

Habitat and activity: The species was observed in forested patches while nectaring.

**Genus *Burara* Swinhoe, 1893**

*Burara jaina* (Moore, 1865) (Fig. 9b).

Common name: Orange awlet.

Identification remarks: UN pale brown. UNH with orange stripes along veins and has orange fringe. UNF purplish. Wingspan 60–70 mm.

Season: Monsoon.

Habitat and activity: The species was observed in forested patches while nectaring.

**Genus *Celaenorrhinus* Hübner, 1819**

*Celaenorrhinus ruficornis* Hampson, 1889 (Fig. 9c).

Common name: Tamil spotted flat.

Identification remarks: Similar to common spotted flat, but UPF has semi-transparent white spots separated from each other. Markings on UPH indistinct or absent. Antennae chequered, club white in male, white at base only in female. Wingspan 45–50 mm.

Season: Monsoon.

Habitat and activity: The species was observed in forested patches while nectaring.

**Genus *Hasora* Moore, 1881**

*Hasora vitta* (Butler, 1870) (Fig. 9d).

Common name: Plain banded awl.

Identification remarks: Outwardly diffused broad white or bluish-white band on UNH. Female has an additional spot on UPF. UN paler, inner half has greenish gloss. Wingspan 45–55 mm.

Season: Monsoon.

Habitat and activity: The species was observed in forested patches while nectaring.

**Genus *Matapa* Moore, 1881**

*Matapa aria* (Moore, 1865) (Fig. 9e).

Common name: Common Redeye.

Identification remarks: Dark buff-brown with no markings on UP. HW has greyish fringe tinged with pale yellow. UN more yellowish orange-brown. Indistinct black brand on UPF of male. Wingspan 40–55 mm.

Season: Monsoon and winter.

Habitat and activity: The species was observed in forested patches while nectaring.

#### Family Lycaenidae Leach, 1815

**Genus *Arhopala* Boisduval, 1832**

*Arhopala amantes* (Hewitson, 1862) (Fig. 9f).

Common name: Large oakblue.

Identification remarks: Tailed with lobe. UNH has central squarish spots in spaces 4 and 5 at right angles. Metallic scales at UNH lower tip. Wingspan 45–57 mm.

Season: Winter.

Habitat and activity: The species was observed in forested patches while mud puddling, basking or feeding on rotten fruits.

*Arhopala centaurus* (Fabricius, 1775) (Fig. 10a).

Common name: Centaur oakblue.

Identification remarks: HW tailed. No HW lobe. Metallic scaling on UNH faint or absent. UNF band continuous and curved. UNF cell spots outlined by silver lines. Male UP brilliant violet-blue, narrow dark borders. Females UP paler blue, broad wing borders. Wingspan 53–62 mm.

Season: Winter.

Habitat and activity: The species was observed in forested patches while mud puddling, basking or feeding on rotten fruits.

**Genus *Cheritra* Moore, 1881**

*Cheritra freja* (Fabricius 1793) (Fig. 10b).

Common name: Common Imperial.

Identification remarks: Two tails. UN of both sexes white to pale brown; faint bars at cell-ends. Narrow dark outer central line on UNF. UNH with outer central and marginal lines and black spots crowned with metallic scales at lower tip. Wingspan 38–42 mm.

Season: Monsoon and winter.

Habitat and activity: The species was observed in forested patches while basking or nectaring.

**Genus *Chliaria* Moore, 1884**

*Chliaria othona* (Hewitson, 1865) (Fig. 10c).

Common name: Orchid Tit.

Identification remarks: Two tails. UN white, faint cell-end bars, black-edged brown markings. UNF band upper part wider than the lower part. UNH central band broken twice; prominent black spot near base. Wingspan 24–27 mm.

Season: Winter.

Habitat and activity: The species was observed in forested patches while mud puddling, basking or nectaring.

**Genus *Spalgis* Moore, 1879**

*Spalgis epius* (Westwood, 1851).

Common name: Apefly (Fig. 10d).

Identification remarks: HW Tailless. UN with several fine wavy vertical lines. Male FW has acute apex and straight outer edge. Female has rounded outer edge. Caterpillars feed on mealy bugs. Wingspan 20–30 mm.

Season: Winter.

Habitat and activity: The species was observed in forested patches while feeding on bird droppings.

**Genus *Spindasis* Donzel, 1847**

*Spindasis vulcanus* (Fabricius, 1775) (Fig. 10e).

Common name: Common silverline.

Identification remarks: Two tails, one lobe on HW. UN light yellow, black or brown bordered brilliant reddish bands with central silver lines. Separate spots at base of UNH and outer basal band of spots does not extend downwards to first costal vein. Orange-crowned black spot on UNH lobe. Female larger than male and with more rounded FW. Wingspan 26–34 mm.

Season: Summer.

Habitat and activity: The species was observed in plains and undulating terrains while either mud puddling, basking, nectaring or feeding on carcass.

**Genus *Tarucus* Moore, 1881**

*Tarucus ananda* (de Nicéville, 1884) (Fig. 10f).

Common name: Dark Pierrot.

Identification remarks: HW Tailed. Resembles Assam Pierrot, differs in having the central spot in space 5 joined to the band of spots near margin on UN. Wingspan 22–28 mm.

Season: Winter.

Habitat and activity: The species was observed in forested patches while mud puddling.

#### Family Nymphalidae Rafinesque, 1815

**Genus *Athyma* Westwood, 1850**

*Athyma inara* Westwood, 1850 (Fig. 11a).

Common name: Colour sergeant.

Identification remarks: UP dark brown with very broad orange bands. In male, UP velvety black with a white band and orange markings. UPF white band continues on UPH. Orange markings on UPF apex. UPH with orange band near outer edge. Wingspan 55–70 mm.

Season: Winter.

Habitat and activity: The species was observed in forested patches while mud puddling or basking.

*Athyma perius* (Linnaeus, 1758) (Fig. 11b)

Common name: Common sergeant.

Identification remarks: A prominent row of black spots always towards the inner edge of the white band on both sides of HW. UPF white cell streak divided into four parts. Wingspan 60–70 mm.

Season: Winter.

Habitat and activity: The species was observed in forested patches while mud puddling or basking.

**Genus *Charaxes* Ochsenheimer, 1816**

*Charaxes psaphon* Westwood, 1847 (Fig. 11c).

Common name: Plain Tawny Rajah.

Identification remarks: Male UN tawny with purple gloss. UPF tawny, broad black terminal border. UPH black terminal broad near apex. Female UN tawny with broad pale central band. UPH tawny with broad black terminal border and central white band. Wingspan 85–110 mm.

Season: Winter.

Habitat and activity: The species was observed in forested patches while mud puddling or basking, feeding on nectar, animal waste or carcasses.

**Genus *Cupha* Billberg, 1820**

*Cupha erymanthis* (Drury, 1773) (Fig. 11d).

Common name: Rustic.

Identification remarks: Basal area of UPF reddish-brown, a broad yellow or white central band and broad black apex. Two darker marginal lines of crescents on UPH. Sexes similar. Wingspan 50–60 mm.

Season: Monsoon and winter.

Habitat and activity: The species was observed in forested patches while mud puddling, basking or nectaring.

**Genus *Euploea* Fabricius, 1807**

*Euploea klugii* Moore, 1858 (Fig. 11e).

Common name: Brown king crow.

Identification remarks: Similar to Common Crow, but UN of either wing has no spots. All wings bordered with series of marginal and sub-marginal white spots. Male has a short, oval, dark band on UPF. UPH has greyish scales on apical half and pale-yellow scent scales patch. Wingspan 85–100 mm.

Season: Summer and winter.

Habitat and activity: The species was observed in forested patches while mud puddling, basking or nectaring.

*Euploea sylvester* (Fabricius, 1793) (Fig. 11f).

Common name: Double branded crow.

Identification remarks: Similar to Common Crow, but male has two parallel brands on UPF; female has two similar faint streaks near inner edge on UPF. Wingspan 95–105 mm.

Season: Summer.

Habitat: The species was observed in forested patches while mud puddling, basking or nectaring.

**Genus *Polyura***

*Polyura bharata* Drury, 1773.

Common name: Cryptic Nawab (Fig. 12a).

Identification remarks: Pale greenish-yellow, wide central band on both sides. Large pale green spot near FW apex on both sides. Wingspan 60–75 mm.

Season: Winter.

Habitat: The species was observed in forested patches while mud puddling or basking, feeding on tree sap, animal waste or carcasses.

**Genus *Tanaecia* Butler, 1869**

*Tanaecia lepidea* (Butler, 1868) (Fig. 12b).

Common name: Grey Count.

Identification remarks: UP dark brown with pale grey border. Border broad on HW and narrow on FW, ending before apex. FW apex produced and outer edge incurved. Female, larger and duller coloured than male, with extra pale brown markings. Wingspan 65–85 mm.

Season: Monsoon and winter.

Habitat and activity: The species was found at forest edges while mud puddling or basking or feeding on tree sap, carcasses, animal waste, bird droppings or rotten fruits.

**Genus *Tirumala* Moore, 1880**

*Tirumala septentrionis* (Butler, 1874) (Fig. 12c).

Common name: Dark Blue Tiger.

Identification remarks: Similar to Blue Tiger, but markings narrower and darker. UNH has a long V-shaped pale blue marking in the cell. UN darker than Blue Tiger. Male UNH has scent scales pouch. Wingspan 75–95 mm.

Season: Summer and winter.

Habitat and activity: The species was observed in forested patches while mud puddling, basking or nectaring.

#### Family Papilionidae Latreille, 1802

**Genus *Pachliopta* Reakirt, 1865**

*Pachliopta aristolochiae* (Fabricius, 1775) (Fig. 13a).

Common name: Common Rose.

Identification remarks: HW tailed. UNF black with pale greyish stripes between veins. UNH has large white patch of five elongate spots around end-cell, series of bright red or brownish-red spots on outer edge. Body red. Wingspan 80–110 mm.

Season: Winter.

Habitat and activity: The species was observed at forests edges, scrubs and in grasslands while nectaring.

*Pachliopta hector* (Linnaeus, 1758) (Fig. 13b).

Common name: Crimson rose.

Identification remarks: HW tailed. Markings on both sides similar. Body bright crimson. Female duller, with larger crimson crescents and spots on HW. Wingspan 90–110 mm.

Season: Winter.

Habitat and activity: The species was observed at forests edges, scrubs and in grasslands while nectaring.

**Genus *Papilio* Linnaeus, 1758**

*Papilio helenus* Linnaeus, 1758 (Fig. 13c).

Common name: Red Helen.

Identification remarks: UPH with patch of three creamy white spots. UPH may have marginal series of indistinct red crescents. Wingspan 110–130 mm.

Season: Summer and monsoon.

Habitat and activity: The species was observed in forested patches while nectaring.

#### Family Pieridae Swainson, 1820

**Genus *Appias* Hübner, 1819**

*Appias albina* (Boisduval, 1836) (Fig. 13d).

Common name: Common Albatross.

Identification remarks: Male UPF with dark dusting in apical area and along outer edge, but may be absent. No dark spot on UPF. Pale dull yellow UNH unmarked. Seasonal variation seen in both sexes. In female, UPF apex, leading edge and outer edge bordered with black with four to five white spots near apex. No cell spot. UPH has toothed black border. Wingspan 60–75 mm.

Season: Monsoon and winter.

Habitat and activity: The species was observed in forested patches while nectaring.

*Appias indra* Moore, 1857 (Fig. 13e)

Common name: Plain Puffin

Identification remarks: Male UPF white with apical, outer and leading (half) edges black with two to five apical white spots. Males of northern population have complete row of four or five apical spots on UPF. UPF has black area along outer edge which extends inwards. In female, UPF black, with central white patch and two white spots at apex. UPH with black outer half and dusky grey or white basal half. UNF with broad dark band from leading edge to outer edge. UNH variable. Wingspan 60–70 mm.

Season: Winter.

Habitat and activity: The species was observed in forested patches while nectaring.

*Appias libythea* Fabricius, 1775 (Fig. 13f).

Common name: Striped Albatross.

Identification remarks: Female DSF white, UPF apex and outer edge broadly black and unspotted, leading edge broadly blackened from base to bar at end-cell. UPH with black spots along outer edge. Female WSF much darker, UN white with diffused greyish-brown markings.

Season: Winter.

Habitat and activity: The species was observed at forests edges, scrubs and in grasslands while nectaring.

## Discussion

### Species Richness

Betham (1894) had hoped that someone from Bombay (= Mumbai) would add to his list of 78 butterflies, quoting the fact that there must be many species which still could be obtained from Matheran. It is our honour to fulfil his wish and almost double the list of available butterflies at Matheran 125 years after his publication. Sixty three species of those recorded by us are common to the checklists of Smith (1882), Betham (1894) and Padhye et al. (2013) (Table 6). All the other 77 species are recorded for the first time from the region. Fifteen species recorded by Smith (1882) and three species recorded by Betham (1894) were not recorded during this study (Table 6). Seventeen species were recorded by Smith (1882) and us, but not by Betham (1894), while the same numbers of species were recorded by Betham (1894) and us, but not by Smith (1882). Our list contains all the species recorded by Padhye et al. (2013). Five specific names from Smith (1882) and Betham (1894) could not be traced and are mentioned as ‘Not Found’ in Table 6.

### Seasonal Turnover

The butterfly diversity and distribution is known to be affected by seasons (Brower 1995, Kunte 2000, Tiple et al. 2009). This is especially true in the case of tropical butterflies which may experience extreme wet and dry seasons (Bonebrake et al. 2010). Further, it has also been observed in the case of southern Indian danaine butterflies that they avoid extreme wet and torrential monsoon conditions through longitudinal migration to drier areas (Kunte 2004). The highest number of butterflies in the winter (N = 125), observed during this survey, could be a result of the fact that winters have lower temperature, lower dampness and moderate water availability with no torrential precipitation in and around the study area. We also observe a dry season ‘pocket effect’ (similar to ‘ithomiine pocket’ observed by Vasconcellos-Neto (1991)) in butterflies of the genus *Mycalesis, Lethe, Ypthima* (Family Nymphalidae) and *Celaenorrhinus*, *Taractrocera* and *Spialia* (Family Hesperiidae). These butterflies could be observed in open areas on hill-tops and hill-slopes during monsoon and winter months, but their number becomes less in these areas during the months of summer when they could be observed in dark, shady habitats. We were, however, unable to determine the cause of the high number of hesperiid observations during the monsoon and this needs a detailed behavioural study.

### Spatial Turnover

The patterns for the diversity of butterflies of Matheran are very similar to those of the California Channel Island Birds and Vanuatu Birds, mentioned by Podani and Schmera (2011). High overall similarity for the entire butterfly diversity (Suppl. material 2) and family-wise similarity between the sites (Fig. 8a-e) indicate the possibility of very stable diversity in the area with very low emigration to, or immigration from, surrounding areas. However, a detailed study from surrounding areas would be required to confirm this fact. The high overall similarity between the pairs of study sites (N = 28) also suggests a higher percentage of habitat generalist species surveyed in and around Matheran.

### Colour coding

This novel approach is expected to improve the representation of the data for seasons and activities of the Indian butterflies. We encourage adding more activities and unique colour codes to make this system more universal, uniform and reader friendly. We also recommend its use while uploading records on open databases, such as Butterflies of India (Kunte et al. 2020) and iNaturalist (https://www.inaturalist.org/) for conveying information regarding the seasons and activities of butterflies.

## Conclusions

A total of 140 species of butterflies belonging to six families were recorded from Matheran, India. This list includes 77 new records for Matheran. We observed a strong seasonal variation in butterfly diversity. The maximum diversity (N = 125) of butterflies was recorded during winter, while the least (N = 80) during monsoon. A high similarity of butterfly species composition was observed between the pairs of sites studied, tending towards perfect nestedness. This also emphasises the fact that the butterfly diversity in the region is quite stable and chances of emigration to, or immigration from, surrounding regions are very low. A strong seasonal gradient for activity patterns was not observed; however, we did observe a 'pocket effect' of dry season on butterflies. Butterflies during the dry season tend to aggregate near damp and shady places. Further, we introduce a novel barcode system for denoting seasons and activities of Indian butterflies and hope that this will help butterfly biologists to concisely and effectively present the data.

## Supplementary Material

B7F5EC55-BD5D-5C37-8899-426DCA2A73CD 10.3897/BDJ.8.e54333.suppl1 Supplementary material 1 Percentage matrix fill and percentage contributions from the SDR-simplex analyses of family-wise and overall species richness. Data type Table File: oo_429763.docx https://binary.pensoft.net/file/429763 Sawant, M., Sarang, S., Modak, N.

E51D2D46-C59D-525F-B592-5880AD01F3F3 10.3897/BDJ.8.e54333.suppl2 Supplementary material 2 Similarity-Richness difference-Species replacement simplex plot for overall butterfly diversity of Matheran showing high similarity. Points denote pair of sites (N = 28) Data type Image File: oo_429764.docx https://binary.pensoft.net/file/429764 Sawant, M., Sarang, S., Modak, N.

## Figures and Tables

**Figure 9a.**
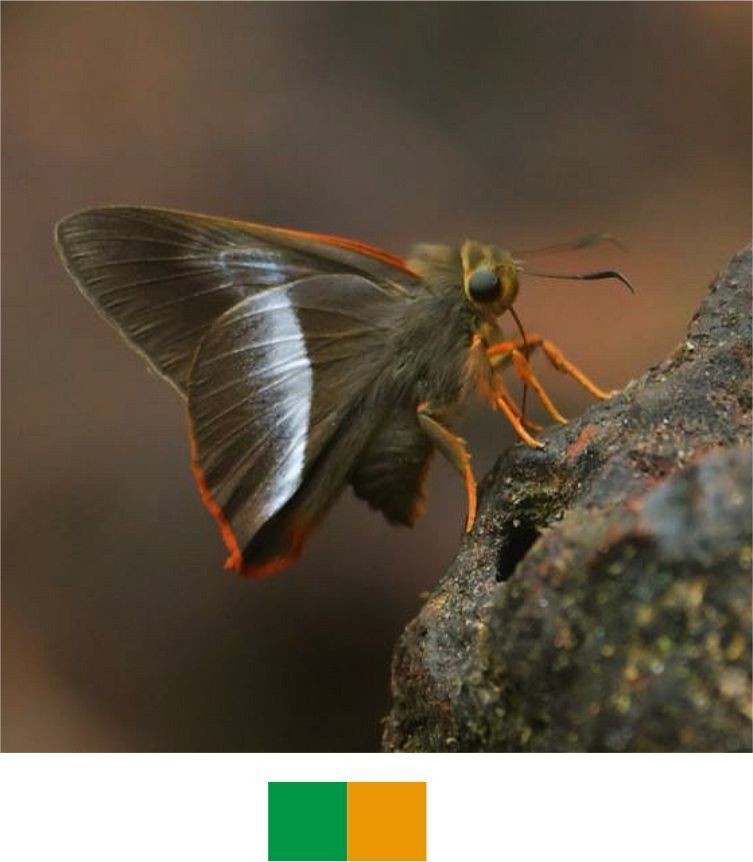
*Bibasis sena*

**Figure 9b.**
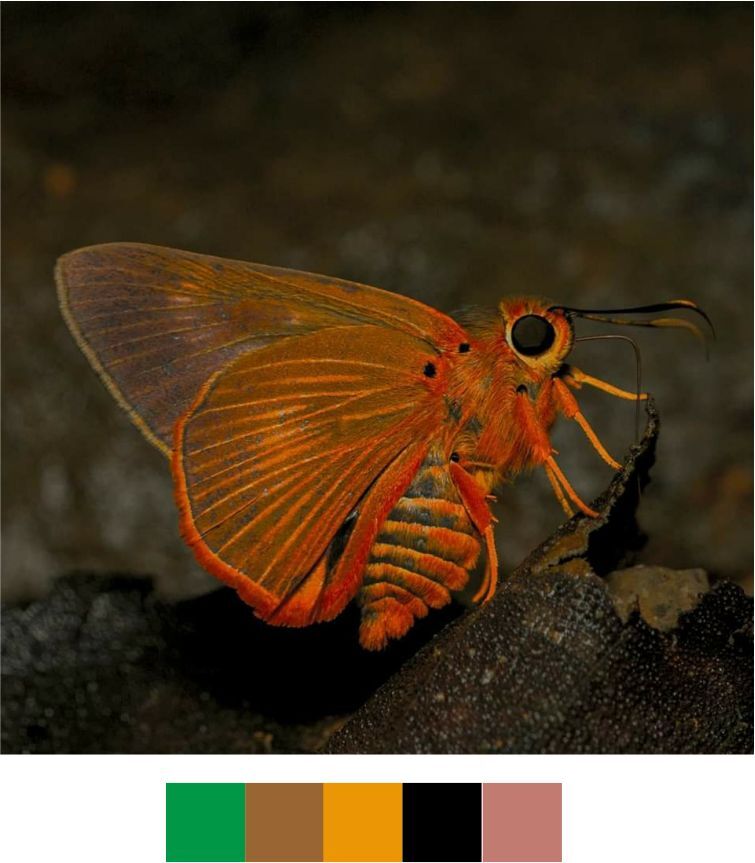
*Burara jaina*

**Figure 9c.**
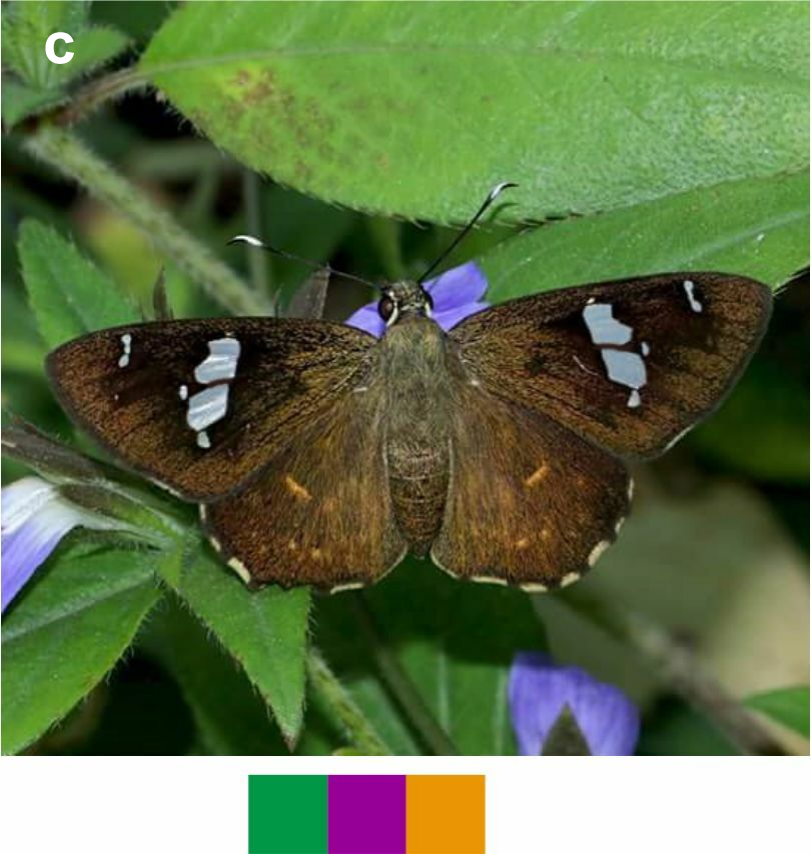
*Celenorrhinus ruficornis*

**Figure 9d.**
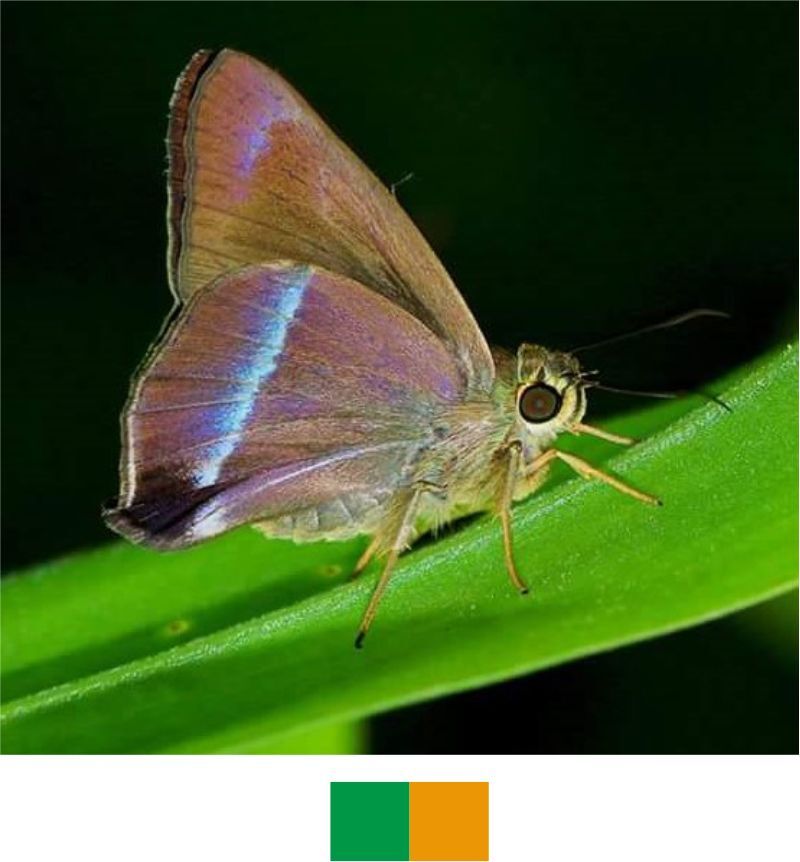
*Hasora vitta* (inverted image)

**Figure 9e.**
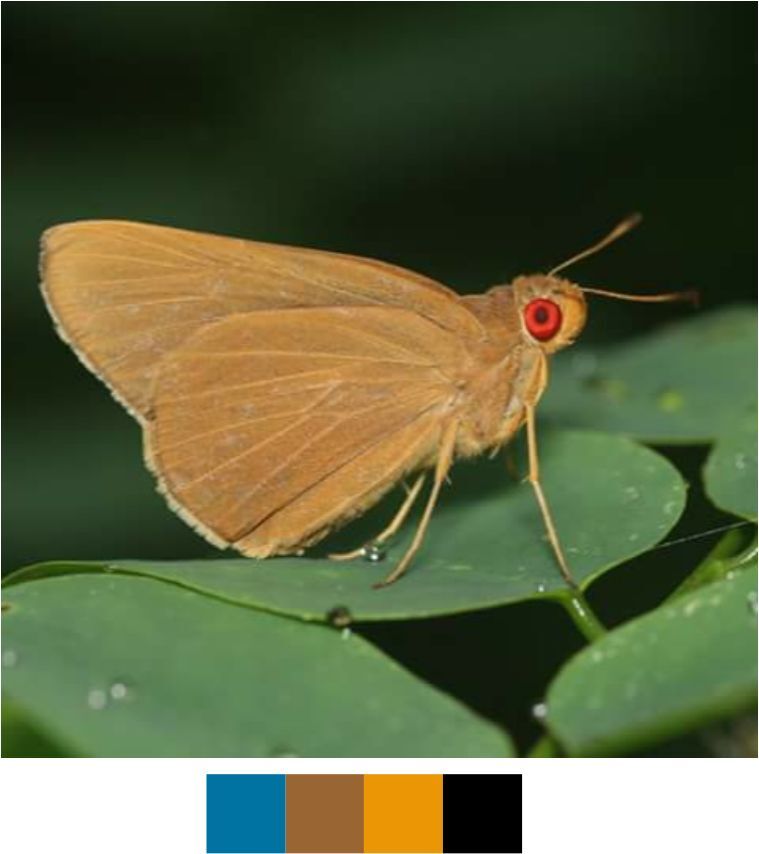
*Matapa aria*

**Figure 9f.**
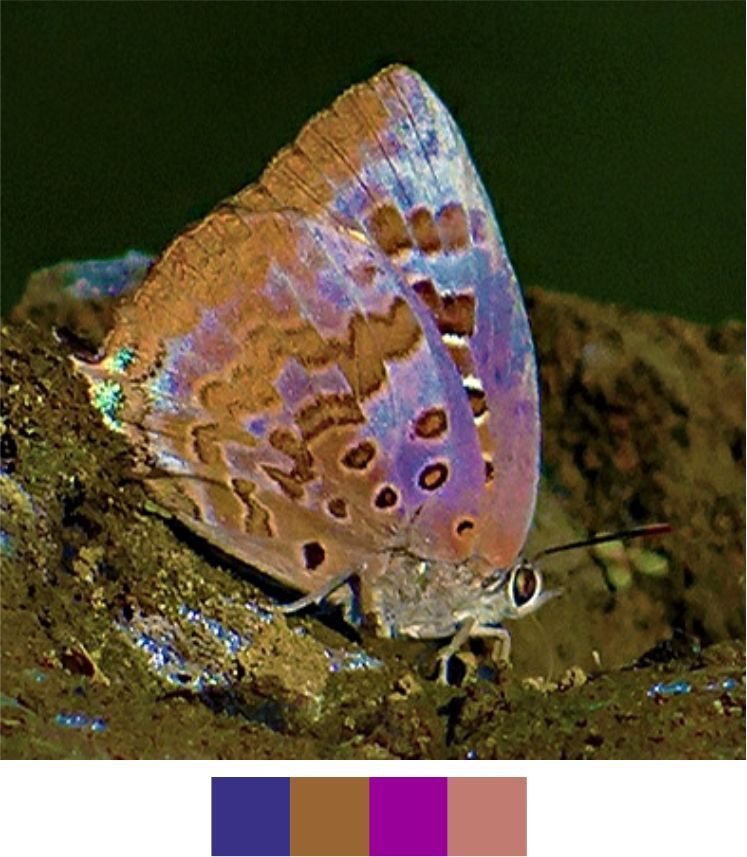
*Arhopala amantes*

**Figure 10a.**
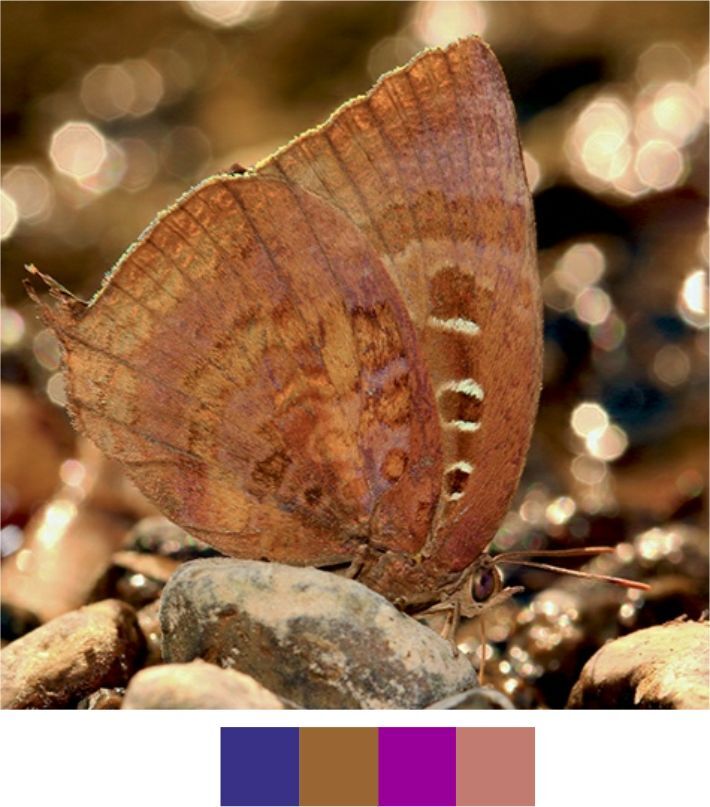
*Arhopala centaurus*

**Figure 10b.**
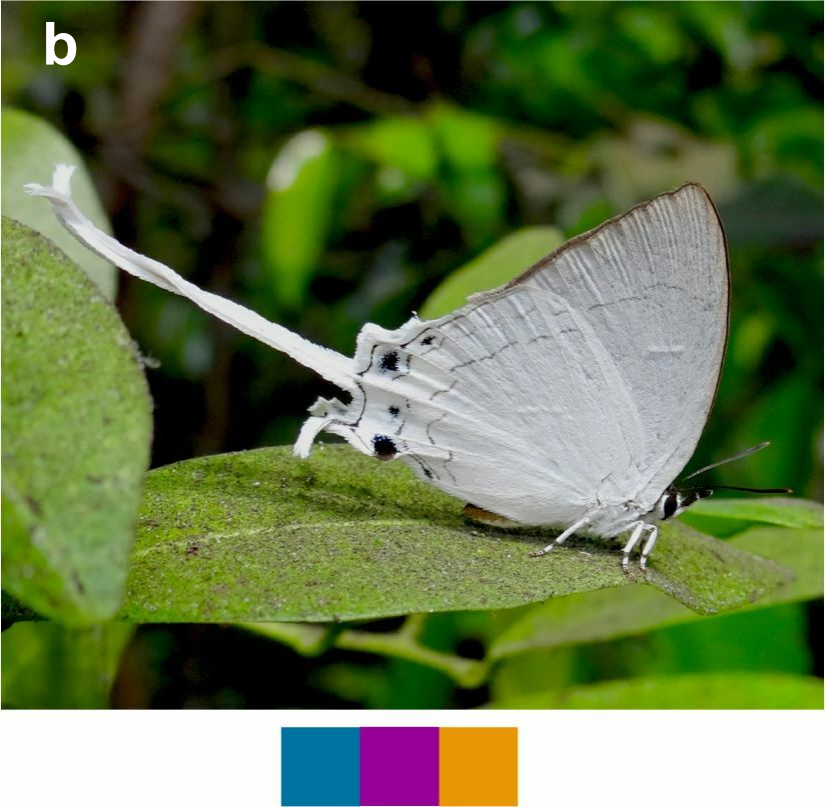
*Cheritra freja*

**Figure 10c.**
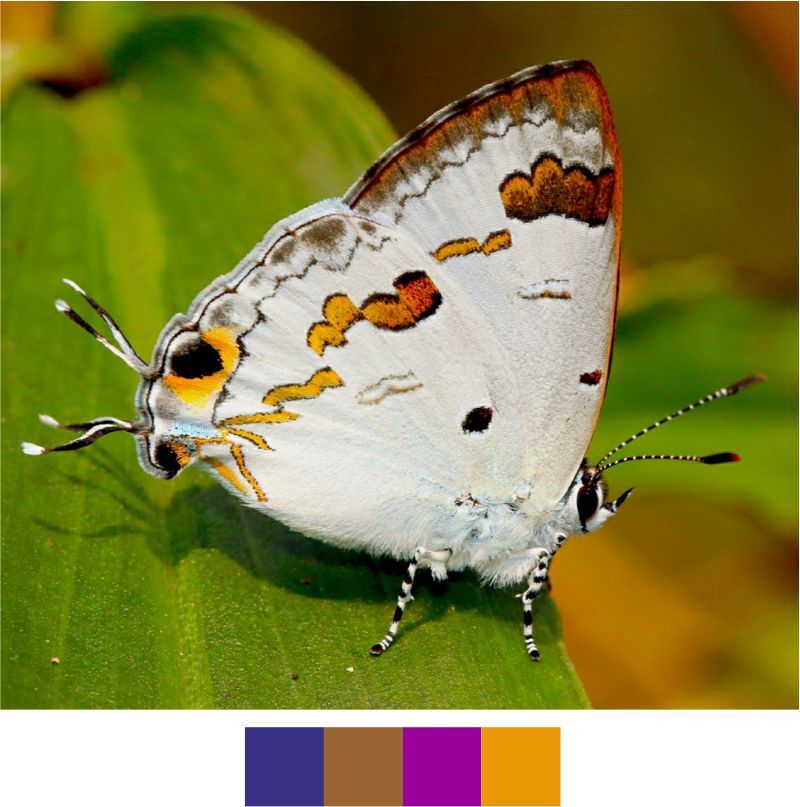
*Chliaria othona*

**Figure 10d.**
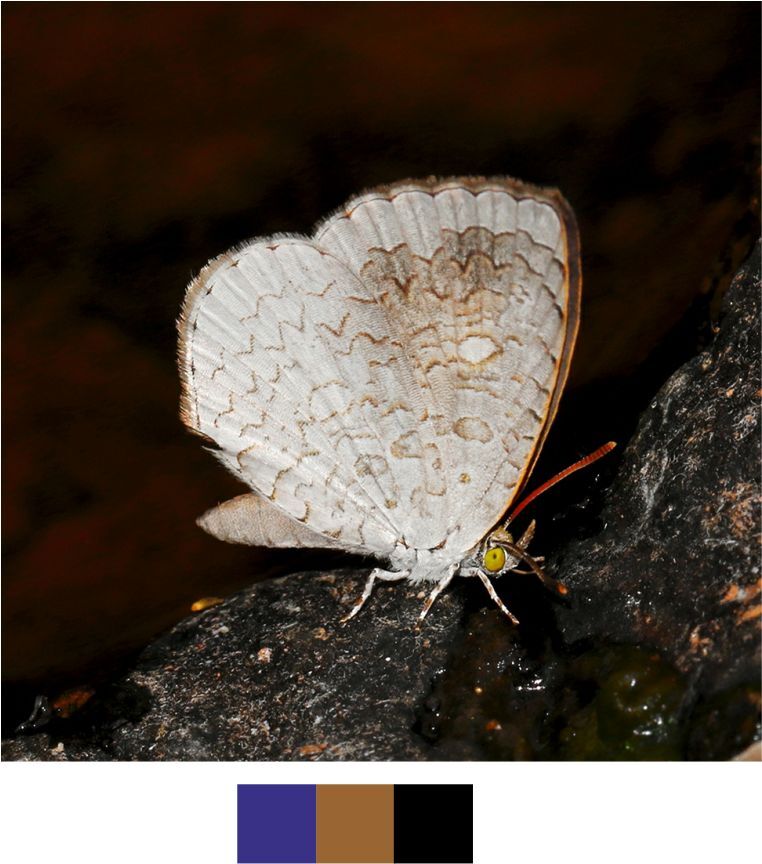
*Spalgis epius*

**Figure 10e.**
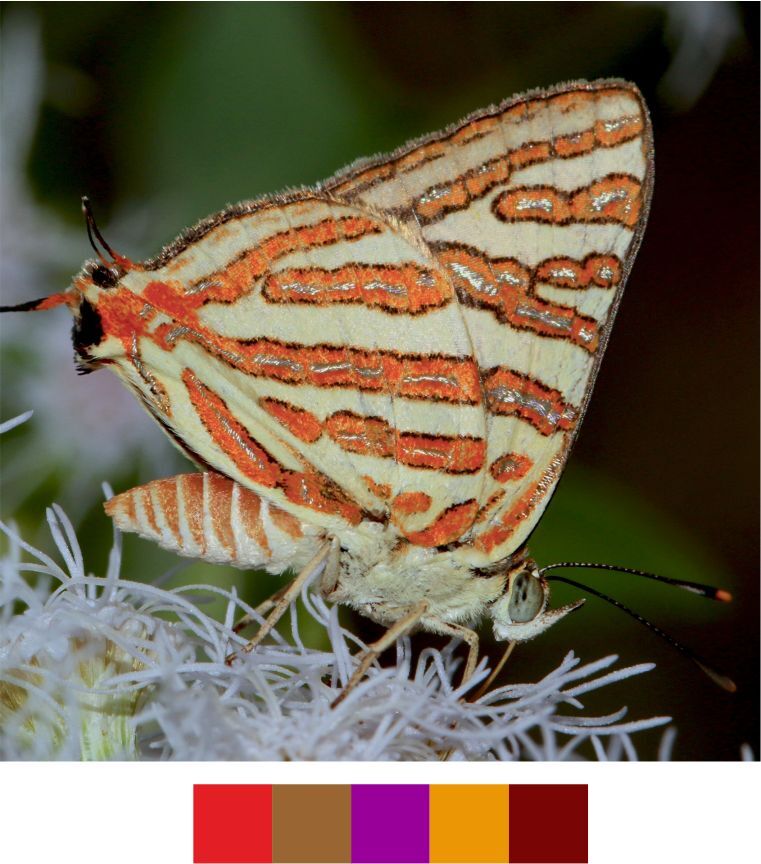
*Spindasis vulcanus*

**Figure 10f.**
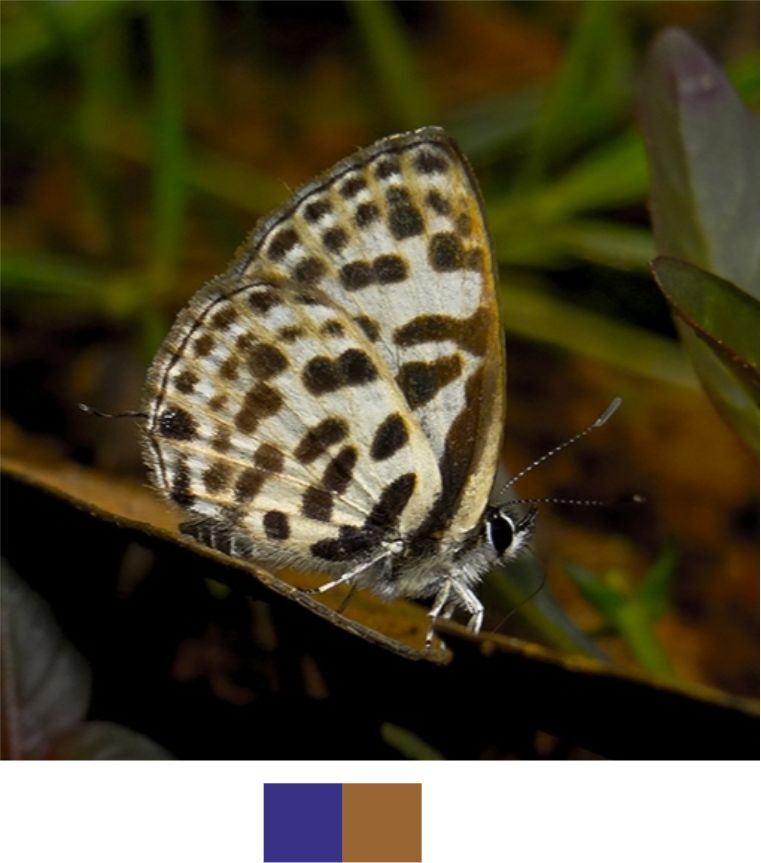
*Tarucus ananda*

**Figure 11a.**
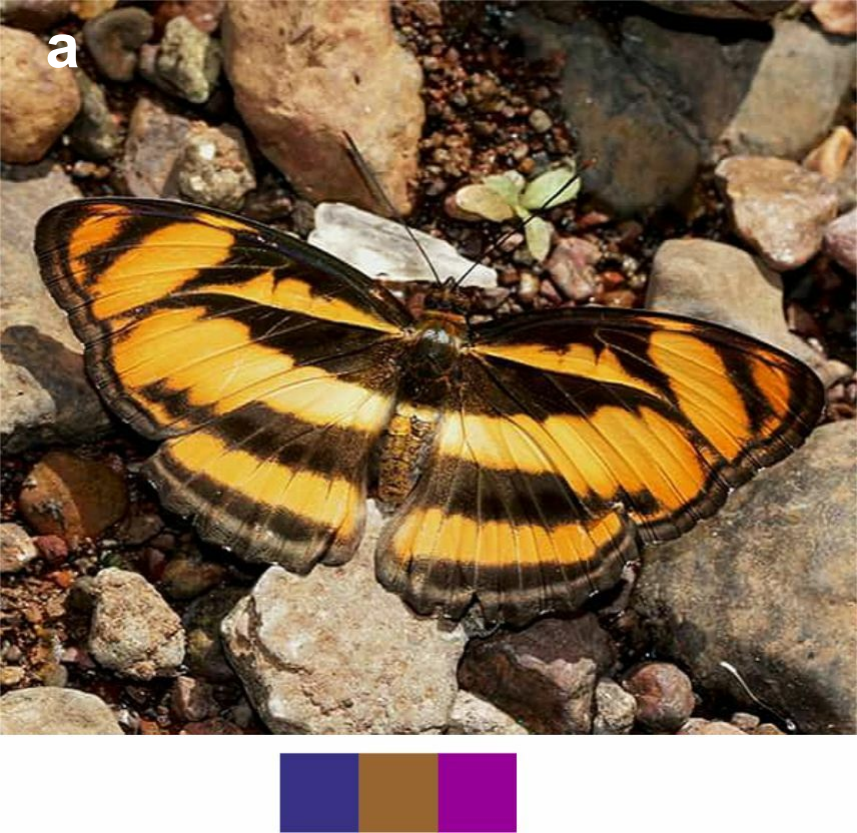
*Athyma inara*

**Figure 11b.**
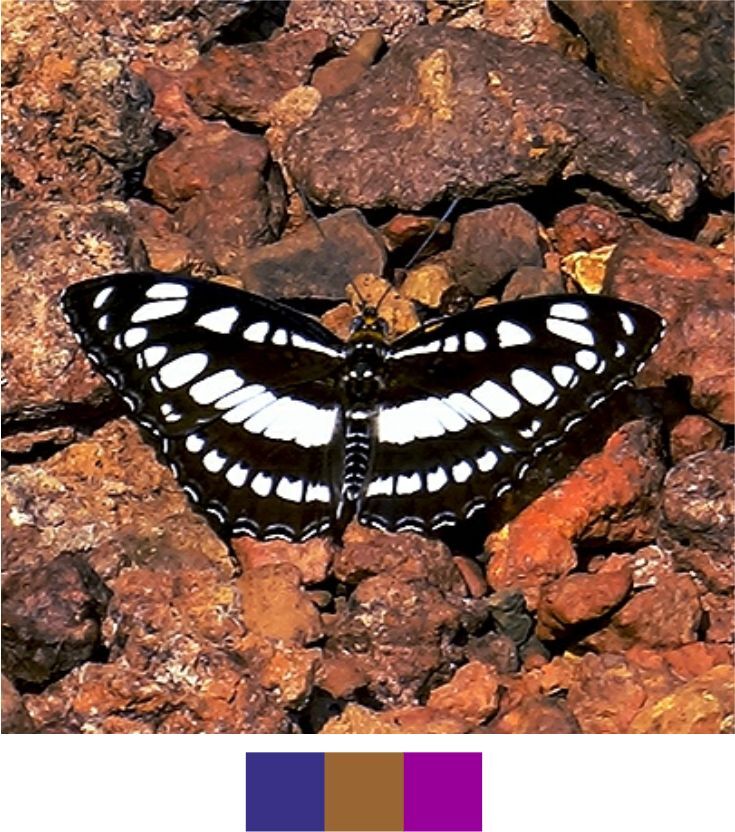
*Athyma perius*

**Figure 11c.**
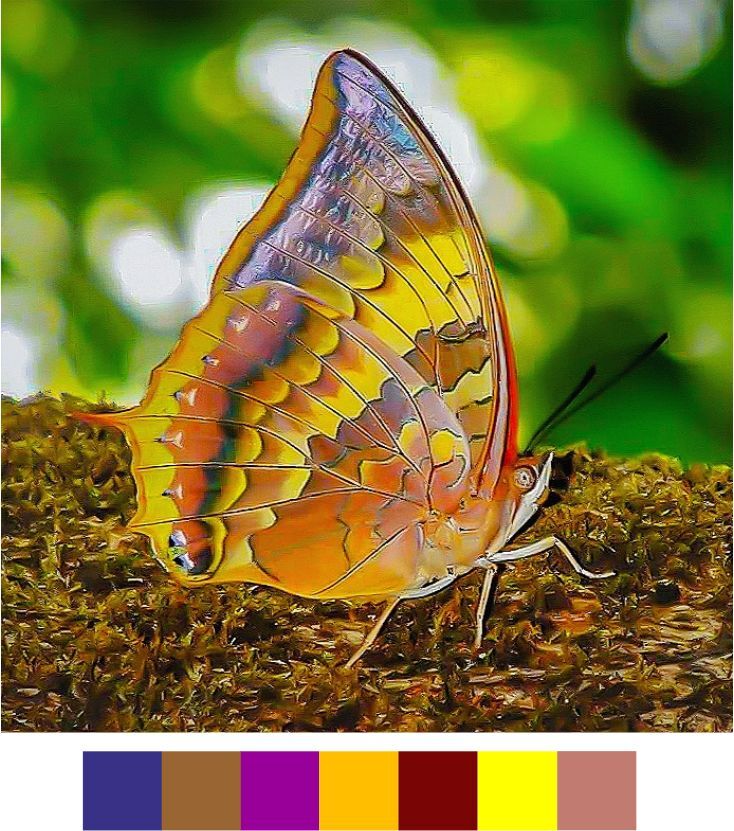
*Charaxes psaphon*

**Figure 11d.**
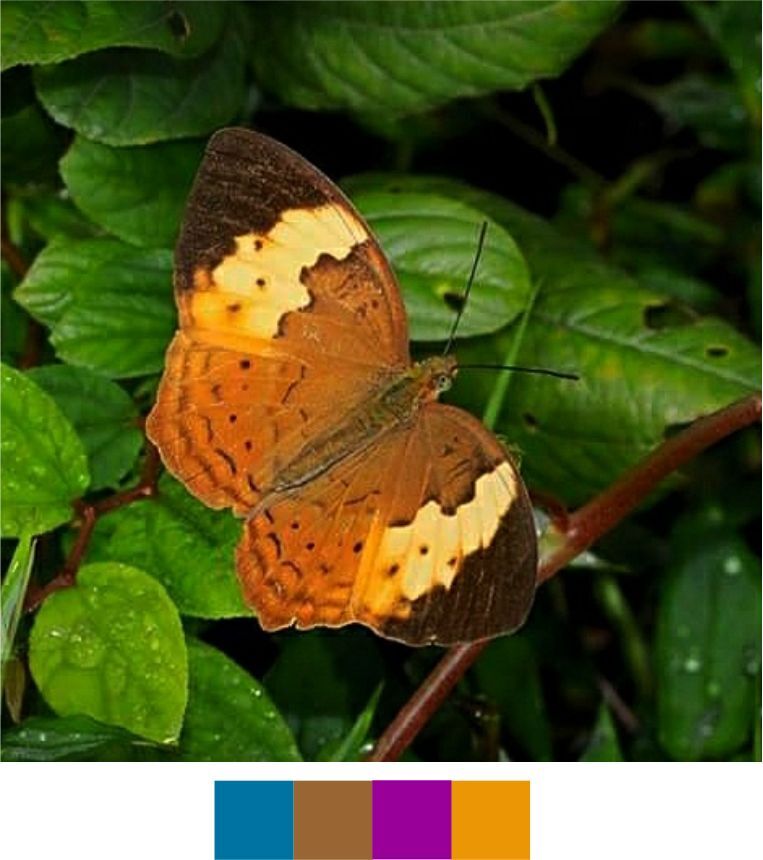
*Cupha erymanthis*

**Figure 11e.**
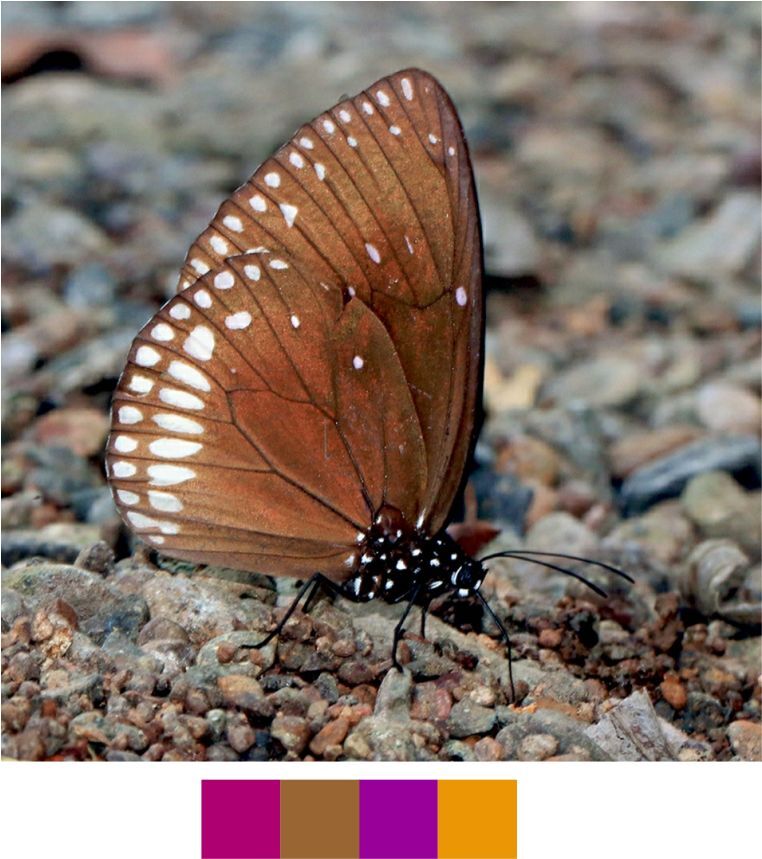
*Euploea klugii*

**Figure 11f.**
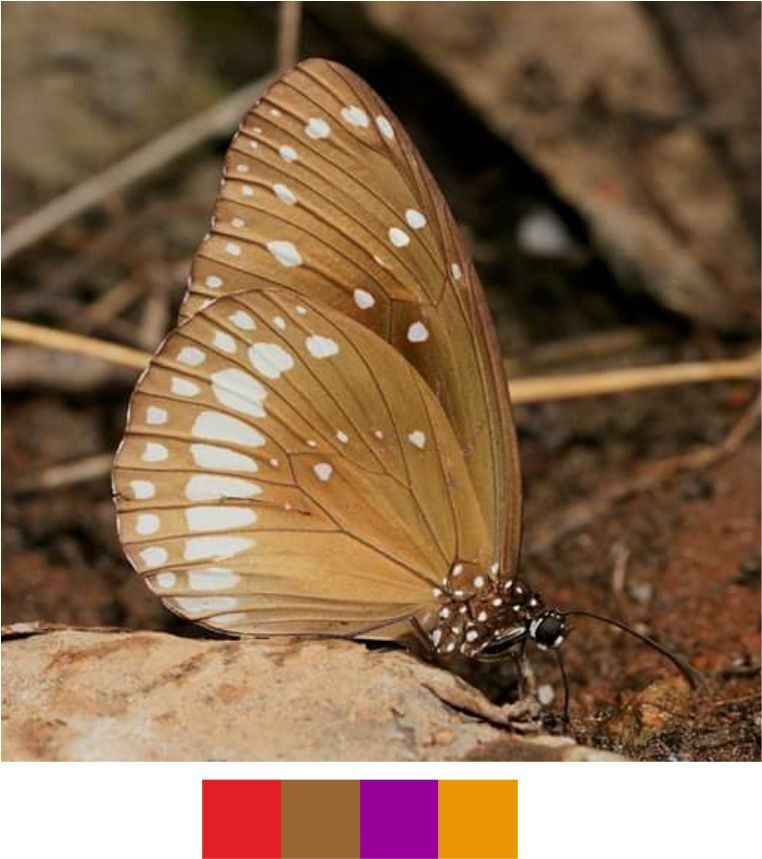
*Euploea sylvester*

**Figure 12a.**
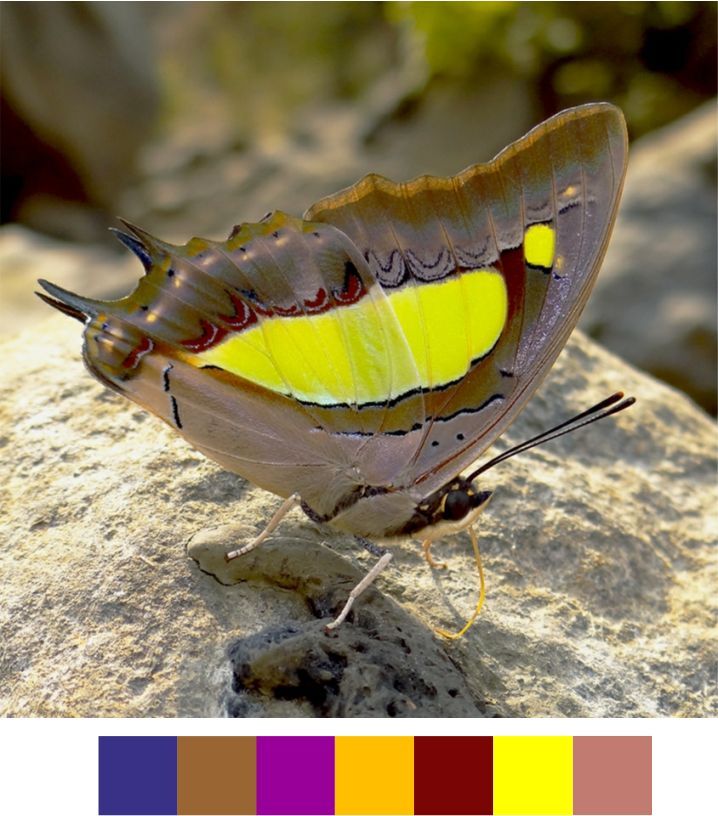
*Polyura bharata*

**Figure 12b.**
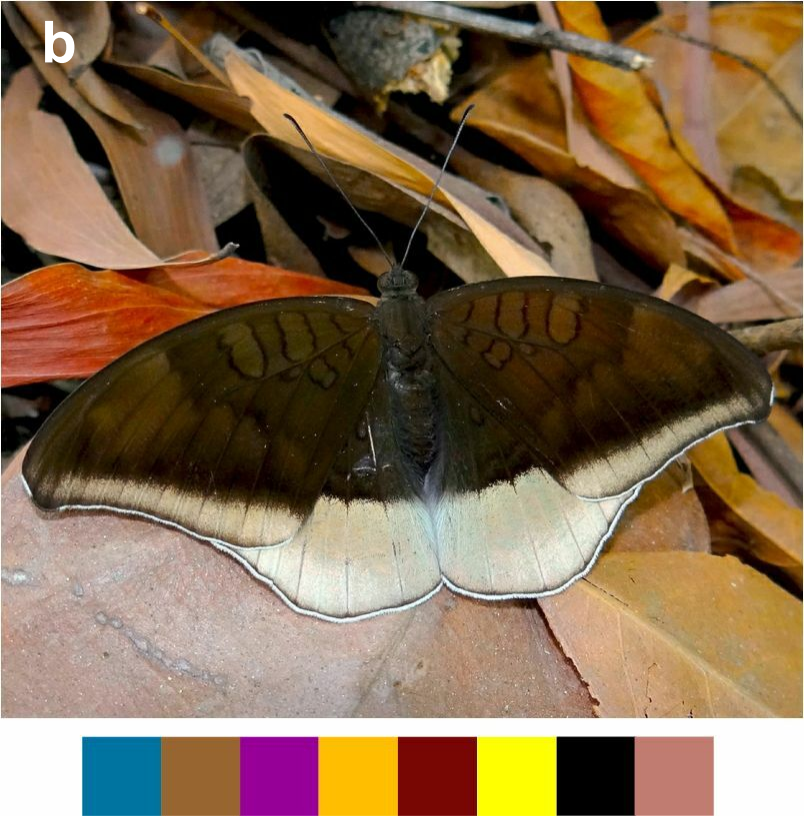
*Tanaecia lepidea*

**Figure 12c.**
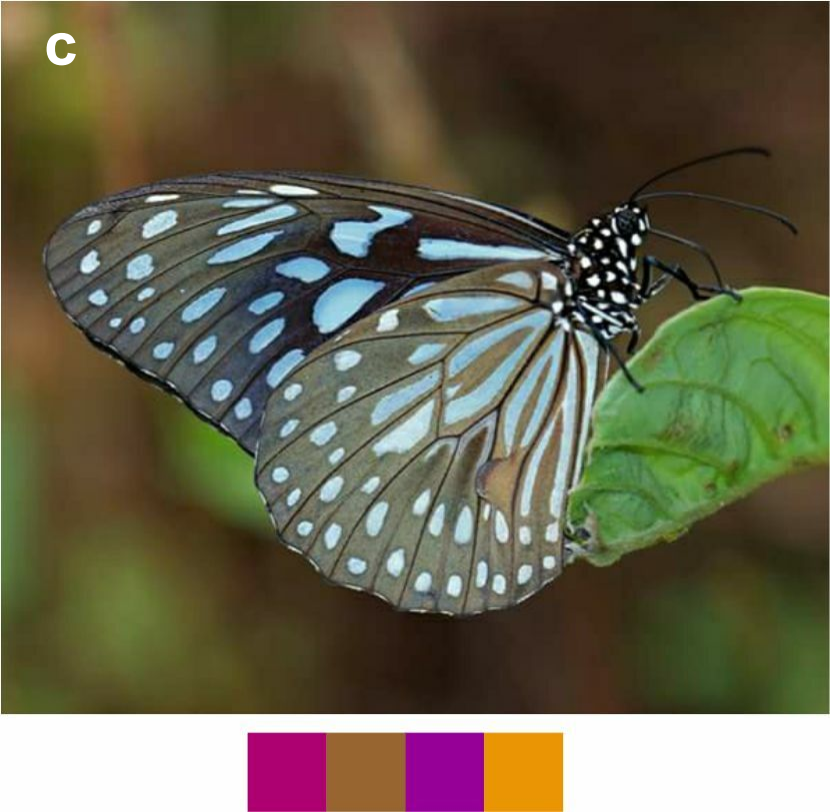
*Tirumala septentrionis*

**Figure 13a.**
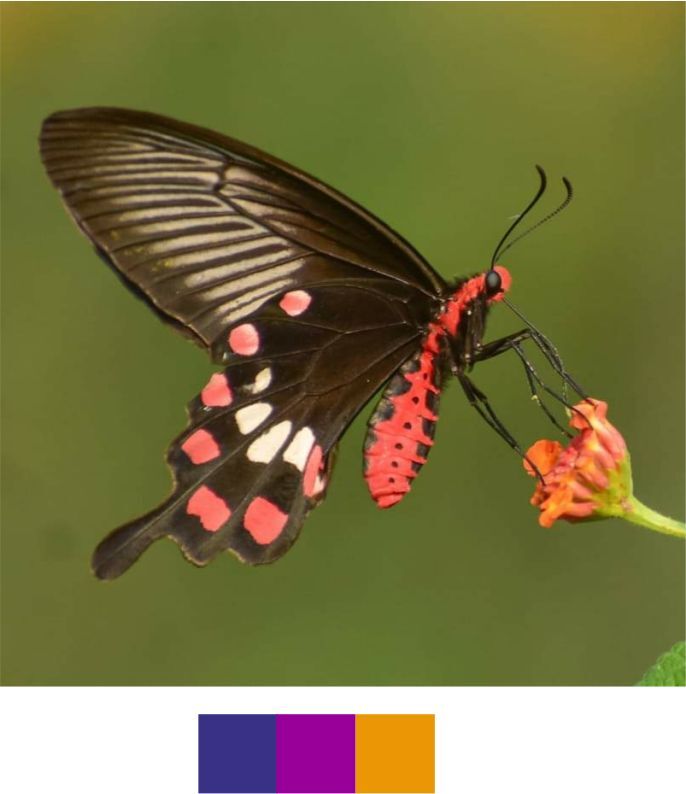
*Pachliopta aristolochiae*

**Figure 13b.**
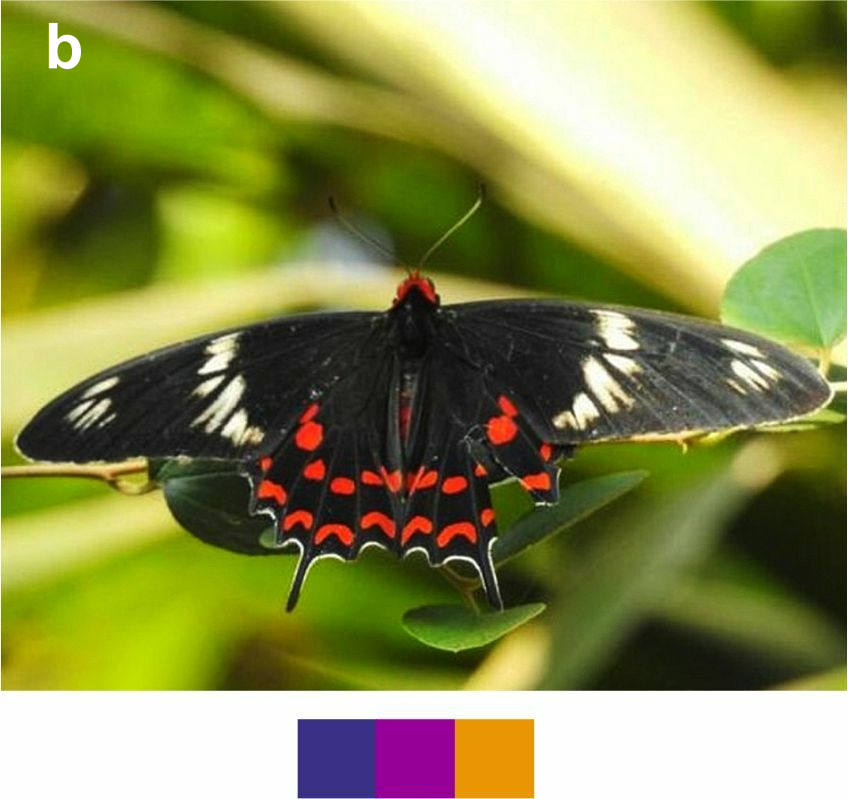
*Pachliopta hector*

**Figure 13c.**
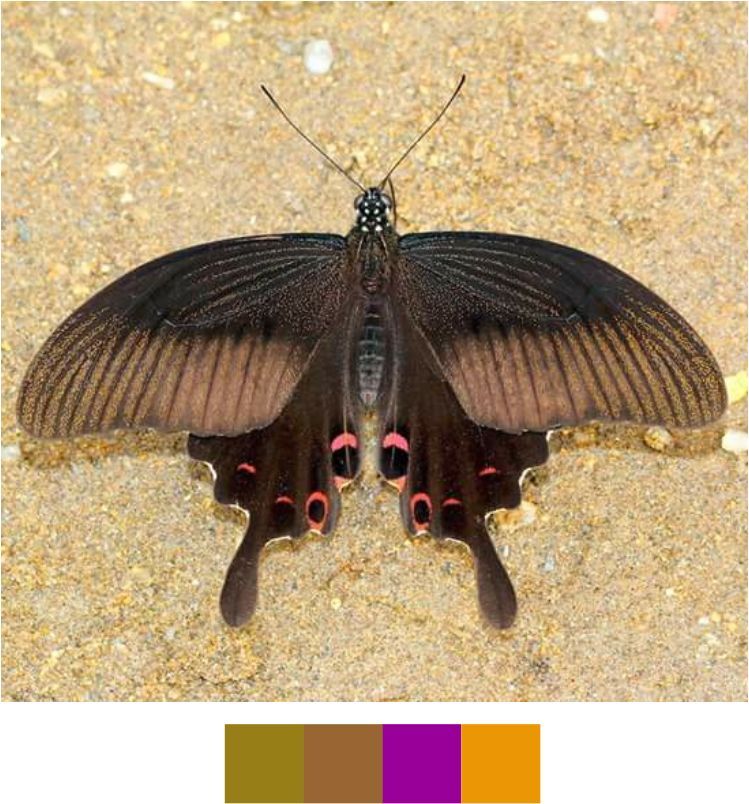
*Papilio helenus*

**Figure 13d.**
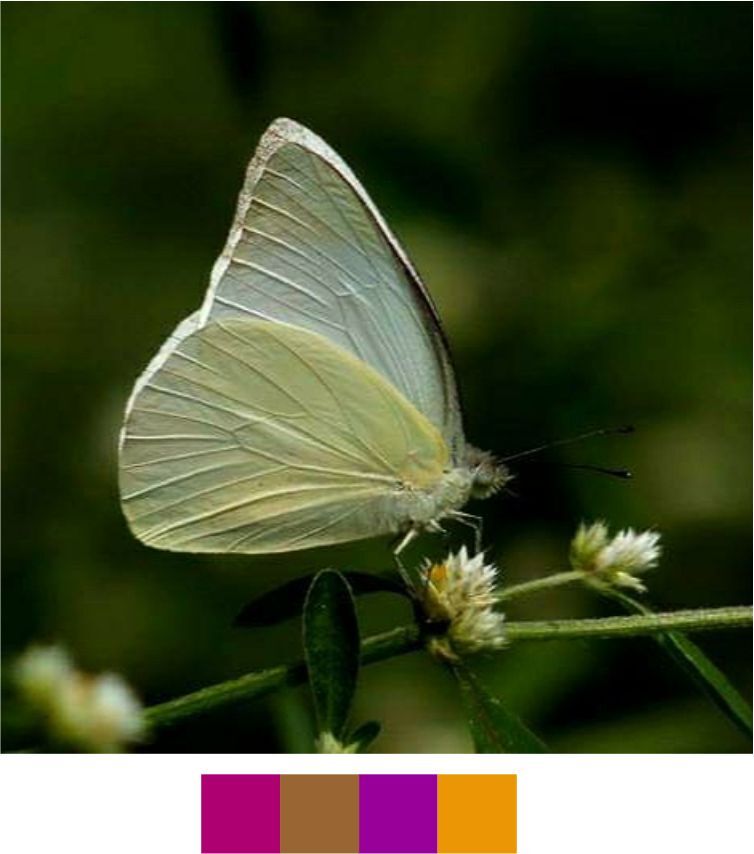
*Appias albina*

**Figure 13e.**
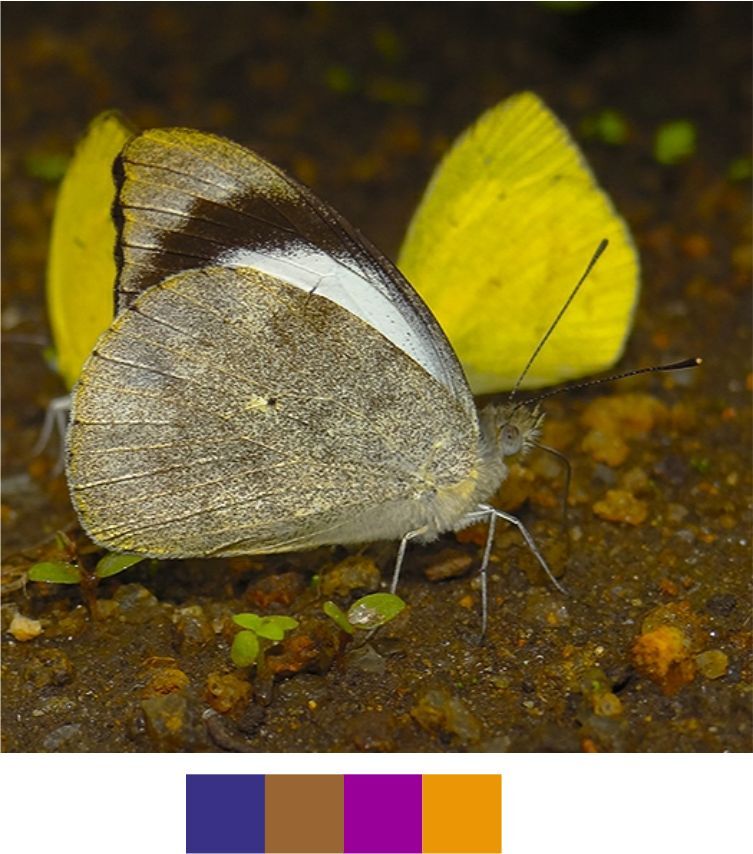
*Appias indra*

**Figure 13f.**
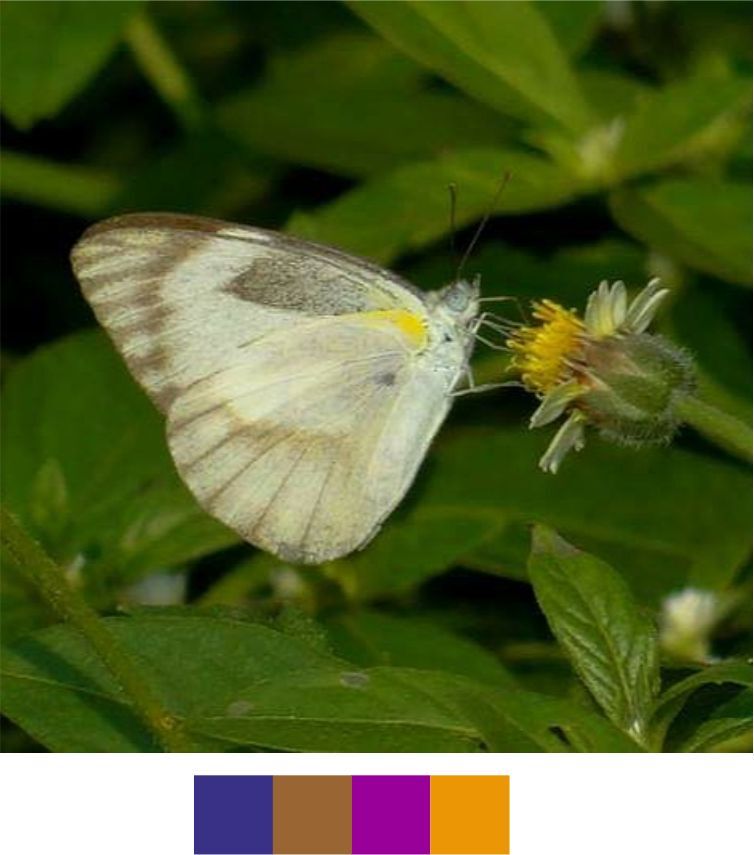
*Appias libythea*

**Table 1.** Survey sites in and around Matheran, India with their geographical, climatic and vegetation characteristics.

**Site code**	**Study area**	**Characteristics**
1	Simpson Tank	Small water barrage built on fast flowing stream surrounded by dense forest. Low canopy cover immediately over the barrage.
2	Charlotte Lake	Large artificial barrage enclosing artificial lake. Surrounded by dense forest.
3	Panorama Point	Mixed vegetation containing semi-evergreen forested patches and grasslands. High ambient moisture during monsoon accompanied by high wind currents.
4	Garbett Point	A small plateau associated with Matheran. Mixed vegetation containing semi-evergreen forested patches and grasslands. A small hamlet sustaining a human population prevalently that of the '*Dhangar*' (Shepherd) tribe.
5	Rustumjee Point	Thick semi-evergreen vegetation. High ambient moisture during monsoon accompanied by high wind currents.
6	One tree hill point	Gradual hill slopes and edge of the valley. Thick semi-evergreen vegetation. High ambient moisture during monsoon accompanied by high wind currents. A torrential stream flows near this area.
7	Neral-Matheran Rail Route	Various types of vegetation elements with patches of wet evergreen, semi-evergreen forests and grasslands. Entire trail has valleys on one side and cliffs on the other. Many torrential streams intersect this area at various points during the monsoon. Cliffs seep with a thin film of water during the monsoon and early winter months. Gutters made for drainage of water hold it until late winter. Shutting down of railway transport during the monsoon leave this area more or less undisturbed from human interference for around four months.
8	Neral-Matheran Road way	Heavily-disturbed area with human interference holding patches of evergreen, semi-evergreen forests, monoculture of *Acacia auriculiformis* and grasslands. Entire trail has valleys on one side and cliffs on the other. Many torrential streams intersect this area at various points during the monsoon. Cliffs seep with a thin film of water during the monsoon and early winter months. Gutters made for drainage of water hold it until late winter and early summer.

**Table 2.** Colour scheme for colour barcodes with CMYK and RGB ratios and HEX numbers.

		**Colour**	**CMYK Ratio (C:M:Y:K)**	**RGB Ratio (R:G:B)**	**HEX**	**Colour Name**
Seasons	Summer		0:100:100:0	227:30:36	#E31E24	Red
	Monsoon		100:0:100:0	0:152:70	#009846	Green
	Winter		100:100:0:0	57:49:133	#393185	Indigo
	Summer+Monsoon		9:24:100:46	151:126:22	#977E16	Tan
	Summer+Winter		24:100:2:13	175:0:113	#AF0071	Purple
	Monsoon+Winter		86:36:9:20	0:115:162	#0073A2	Teal
	Summer+Monsoon+Winter		47:38:38:24	128:128:128	#808080	Grey (50% Black)
	Mud Puddling		19:52:85:37	153:102:51	#996633	Brown
	Basking		57:100:0:0	153:0:153	#990099	Magenta
Feeding	Nectaring		0:45:10:4	235:150:5	#EB9605	Honey (Orange)
	Tree Sap		0:28:98:0	255:191:0	#FFBF00	Amber
	Animal Carcass		11:99:100:50	121:06:04	#790604	Kryon Cherry Red
	Animal Waste		3:0:93:0	255:255:0	#FFFF00	Yellow
	Bird Droppings		95:95:45:95	0:00:00	#000000	Black
	Rotten fruits		17:56:48:12	193:123:113	#C17B71	Rose Brown

**Table 3.** List of butterflies of Matheran. Numeric codes of sites correspond to Table 1. Colour codes of season/s correspond to Table 2. VC- Very Common, C- Common, NC - Not Common, R - Rare, VR - Very Rare. Presence = 1; Absence = 0.

**Common Name**	**Scientific Name**	**Season**	**Local Status**	**Study Sites**							
				**1**	**2**	**3**	**4**	**5**	**6**	**7**	**8**
**Family: Hesperiidae (N = 25)**											
Vindhyan Bob	*Arnetta vindhiana*	All	VC	1	1	1	1	1	1	1	1
Brown Awl	*Badamia exclamationis*	All	C	1	1	1	1	1	1	1	1
Orange-Tailed Awlet	*Bibasis sena*	Monsoon	VR	0	0	1	0	1	1	0	0
Orange Awlet	*Burara jaina*	Monsoon	VR	0	1	1	0	0	1	0	0
Blank Swift	*Caltoris kumara*	Monsoon	VC	0	1	1	1	1	1	1	1
Golden Angle	*Caprona ransonnetii*	All	C	1	0	1	1	1	0	1	0
Malabar Flat	*Celaenorrhinus ambareesa*	All	VC	1	1	1	1	1	1	1	1
Common Spotted Flat	*Celaenorrhinus leucocera*	All	VC	1	1	1	1	1	1	1	1
Tamil Spotted Flat	*Celaenorrhinus ruficornis*	Monsoon	VR	0	0	0	1	0	1	0	0
Tricolor Pied Flat	*Coladenia indrani*	Monsoon+Winter	VC	1	0	1	1	1	1	1	1
Common Awl	*Hasora badra*	Winter	NC	0	1	1	1	1	1	0	0
Common Banded Awl	*Hasora chromus*	All	VC	0	1	1	1	1	1	1	1
Plain Banded Awl	*Hasora vitta*	Monsoon	VR	0	1	1	0	1	0	0	0
Chestnut Bob	*Iambrix salsala*	All	VC	1	1	1	1	1	1	1	1
Common Redeye	*Matapa aria*	Monsoon+Winter	R	1	0	1	1	0	1	0	0
Conjoined Swift	*Pelopidas conjuncta*	Monsoon	VC	1	1	1	1	0	1	1	1
Variable Swift	*Pelopidas mathias*	Monsoon+Winter	C	1	0	1	1	0	1	0	0
Common Small Flat	*Sarangesa dasahara*	All	VC	1	1	1	1	1	1	1	1
Spotted Small Flat	*Sarangesa purendra*	All	VC	1	1	1	1	1	1	1	1
Indian Skipper	*Spialia galba*	Monsoon	C	0	0	1	0	0	1	1	1
Indian Palm Bob	*Suastus gremius*	Winter	C	0	1	1	1	1	1	0	0
Black Angle	*Tapena thwaitesi*	Monsoon+Winter	C	1	0	1	1	1	1	1	0
Tamil Grass Dart	*Taractrocera ceramas*	Summer+Monsoon	VC	0	1	1	1	1	1	1	1
Dark Palm Dart	*Telicota bambusae*	All	C	1	1	1	1	1	1	1	1
Grass Demon	*Udaspes folus*	Monsoon+Winter	C	1	0	1	1	0	1	1	0
**Family: Lycaenidae (N = 46)**											
Common Hedge Blue	*Acytolepis puspa*	All	VC	1	1	1	1	1	1	0	1
Purple Leaf Blue	*Amblypodia anita*	Summer+Winter	C	1	1	1	1	1	0	0	0
Pointed Ciliate Blue	*Anthene lycaenina*	All	VC	1	1	1	1	1	0	0	1
Large Oakblue	*Arhopala amantes*	Winter	VR	1	0	0	1	1	0	0	0
Centaur Oakblue	*Arhopala centaurus*	Winter	VR	1	0	1	0	1	0	0	0
Angled Pierrot	*Caleta decidia*	All	VC	1	0	1	1	1	0	0	1
Common Pierrot	*Castalius rosimon*	All	VC	1	1	1	1	1	1	0	1
Forgetmenot	*Catochrysops strabo*	All	VC	1	1	1	1	1	1	1	1
Common Imperial	*Cheritra freja*	Monsoon+Winter	VR	0	0	0	1	1	1	1	0
Lime Blue	*Chilades lajus*	Summer+Winter	NC	1	0	0	1	1	0	0	0
Orchid Tit	*Chliaria othona*	Winter	VR	1	0	0	0	0	0	0	0
Angled Sunbeam	*Curetis dentata*	Summer+Winter	C	1	0	0	1	1	0	1	0
Indian Sunbeam	*Curetis thetis*	Monsoon+Winter	C	1	0	0	0	1	0	0	0
Cornelian	*Deudorix epijarbas*	All	C	0	0	1	1	1	1	1	1
Gram Blue	*Euchrysops cnejus*	Summer+Winter	C	1	1	1	1	1	0	0	0
Indian Cupid	*Everes lacturnus*	Summer+Winter	NC	1	0	1	1	0	0	0	0
Small Grass Jewel	*Freyeria putli*	Summer+Winter	C	0	1	1	1	0	0	1	1
Silverstreak Blue	*Iraota timoleon*	Summer+Winter	VC	1	0	1	1	1	1	0	0
Dark Cerulean	*Jamides bochus*	All	VC	1	1	1	1	1	1	1	1
Common Cerulean	*Jamides celeno*	All	VC	1	1	1	1	1	1	1	1
Peablue	*Lampides boeticus*	Winter	C	1	0	1	1	1	1	0	1
Zebra Blue	*Leptotes plinius*	Summer+Winter	C	1	0	1	1	1	0	1	1
Yamfly	*Loxura atymnus*	Monsoon+Winter	NC	0	1	1	1	1	1	0	0
Plains Cupid	*Luthrodes pandava*	Winter	C	1	1	0	1	1	0	0	0
Malayan	*Megisba malaya*	Winter	C	1	0	0	0	1	1	0	1
Opaque Six Lineblue	*Nacaduba beroe*	Summer+Winter	VC	1	0	0	0	1	1	0	0
Transparent Six Lineblue	*Nacaduba kurava*	Summer+Winter	VC	1	0	0	0	1	1	0	0
Dingy Lineblue	*Petrelaea dana*	Winter	C	1	0	0	0	1	0	0	0
Tailless Lineblue	*Prosotas dubiosa*	Summer+Winter	VC	1	1	1	1	1	1	0	1
Common Lineblue	*Prosotas nora*	Summer+Winter	VC	1	1	1	1	1	1	0	1
Common Red Flash	*Rapala iarbus*	Summer+Winter	C	1	1	1	1	1	0	0	1
Slate Flash	*Rapala manea*	Summer+Winter	VC	1	1	1	1	1	1	0	1
Indigo Flash	*Rapala varuna*	Summer+Winter	VC	1	0	0	0	1	0	0	0
Monkey Puzzle	*Rathinda amor*	All	VC	0	1	1	1	1	1	1	0
Common Apefly	*Spalgis epius*	Winter	VR	1	0	0	0	0	0	0	0
Long Banded Silverline	*Spindasis lohita*	Winter	NC	1	0	1	1	1	0	0	0
Common Silverline	*Spindasis vulcanus*	Summer	VR	0	1	1	1	1	0	0	0
Common Acacia Blue	*Surendra quercetorum*	Monsoon	NC	0	0	1	1	1	1	1	0
Peacock Royal	*Tajuria cippus*	Winter	C	1	0	0	0	1	1	0	0
Red Pierrot	*Talicada nyseus*	Summer+Winter	C	0	0	1	1	1	0	0	1
Dark Pierrot	*Tarucus ananda*	Winter	VR	0	0	0	0	1	0	0	0
Common Guava Blue	*Virachola isocrates*	All	C	1	0	0	0	1	1	0	0
Large Guava Blue	*Virachola perse*	All	VC	1	0	0	0	1	1	0	0
Dark Grass Blue	*Zizeeria karsandra*	All	VC	1	1	1	1	1	1	1	1
Lesser Grass Blue	*Zizina otis*	All	VC	1	1	1	1	1	1	1	1
Tiny Grass Blue	*Zizula hylax*	Summer+Winter	VC	1	1	1	1	1	1	1	1
**Family: Nymphalidae (N = 44)**											
Angled Castor	*Ariadne ariadne*	All	C	0	1	1	1	1	1	1	1
Common Castor	*Ariadne merione*	All	NC	1	1	1	1	1	1	1	1
Color Sergeant	*Athyma inara*	Winter	VR	1	0	0	0	1	1	1	0
Common Sergeant	*Athyma perius*	Winter	VR	1	0	0	0	1	1	1	0
Tawny Rajah	*Charaxes psaphon*	Winter	R	1	1	1	1	0	0	1	0
Black Rajah	*Charaxes solon*	Winter	NC	1	0	0	1	1	0	0	0
Rustic	*Cupha erymanthis*	Monsoon+Winter	VR	1	0	1	0	1	1	1	0
Common Map	*Cyrestis thyodamas*	Summer+Winter	NC	1	0	1	0	1	1	1	1
Plain Tiger	*Danaus chrysippus*	All	C	1	1	1	1	1	1	1	1
Striped Tiger	*Danaus genutia*	All	C	1	1	1	1	1	1	1	1
Common Crow	*Euploea core*	All	C	1	1	1	1	1	1	1	1
Brown King Crow	*Euploea klugii*	Summer+Winter	R	1	0	1	1	1	0	0	0
Double Branded Crow	*Euploea sylvester*	Summer	VR	1	0	0	0	0	0	0	0
Common Baron	*Euthalia aconthea*	All	VC	1	1	1	1	1	0	1	1
Gaudy Baron	*Euthalia lubentina*	Winter	C	1	1	1	1	1	0	1	1
Great Eggfly	*Hypolimnas bolina*	All	C	1	1	1	1	1	1	1	1
Danaid Eggfly	*Hypolimnas misippus*	All	VC	1	1	1	1	1	1	1	1
Peacock Pansy	*Junonia almana*	All	C	1	1	1	1	1	1	1	1
Grey Pansy	*Junonia atlites*	Summer	NC	1	1	1	1	1	1	1	1
Chocolate Pansy	*Junonia iphita*	All	C	1	1	1	1	1	1	1	1
Lemon Pansy	*Junonia lemonias*	All	C	1	1	1	1	1	1	1	1
Blue Oakleaf	*Kallima horsfieldii*	Monsoon+Winter	C	1	1	1	1	1	1	1	0
Bamboo Treebrown	*Lethe europa*	All	NC	0	1	1	0	1	1	1	0
Common Treebrown	*Lethe rohria*	All	VC	0	1	1	0	1	1	1	1
Club Beak	*Libythea myrrha*	Winter	NC	1	0	0	0	1	0	1	0
Common Evening Brown	*Melanitis leda*	All	VC	1	1	1	1	1	1	1	1
Commander	*Moduza procris*	All	VC	1	1	1	1	1	1	1	1
Dark Brand Bushbrown	*Mycalesis mineus*	Monsoon+Winter	NC	1	1	1	0	1	0	0	0
Common Bushbrown	*Mycalesis perseus*	Monsoon+Winter	C	1	1	1	1	1	1	1	1
Long Brand Bushbrown	*Mycalesis visala*	Monsoon+Winter	VC	1	1	1	0	1	0	0	0
Common Sailer	*Neptis hylas*	All	C	1	1	1	1	1	1	1	1
Chestnut Streaked Sailer	*Neptis jumbah*	Winter	C	1	1	1	0	1	0	0	1
Glassy Tiger	*Parantica aglea*	Monsoon+Winter	C	1	1	1	1	1	1	1	1
Short Banded Sailer	*Phaedyma columella*	Winter	NC	1	0	0	0	1	0	0	0
Common Leopard	*Phalanta phalantha*	All	VC	1	1	1	1	1	1	1	1
Cryptic Nawab	*Polyura bharata*	Winter	R	0	0	1	1	0	0	0	0
Black Prince	*Rohana parisatis*	Summer+Winter	C	1	0	0	0	1	1	1	0
Baronet	*Symphaedra nais*	Summer+Winter	NC	1	0	1	1	0	0	0	0
Grey Count	*Tanaecia lepidea*	Monsoon+Winter	R	0	0	0	0	1	1	1	0
Blue Tiger	*Tirumala limniace*	Monsoon	C	1	1	1	1	1	1	1	1
Dark Blue Tiger	*Tirumala septentrionis*	Summer+Winter	R	1	0	1	1	1	0	0	0
Painted Lady	*Vanessa cardui*	All	C	0	1	1	1	1	1	1	1
Common Fivering	*Ypthima baldus*	All	C	1	1	1	1	1	1	1	1
Common Fourring	*Ypthima huebneri*	All	C	1	1	1	1	1	1	1	1
**Family: Papilionidae (N = 10)**											
Tailed Jay	*Graphium agamemnon*	Monsoon	VC	1	1	1	1	1	1	1	1
Common Jay	*Graphium doson*	Summer+Winter	C	1	1	1	1	1	0	0	1
Bluebottle	*Graphium teredon*	Winter	C	1	0	1	1	1	0	0	1
Common Rose	*Pachliopta aristolochiae*	Winter	R	0	1	0	1	1	0	1	0
Crimson Rose	*Pachliopta hector*	Winter	R	0	1	1	1	1	0	1	0
Common Mime	*Papilio clytia*	Winter	NC	1	0	1	1	1	0	0	0
Lime	*Papilio demoleus*	Summer+Winter	NC	0	1	1	1	1	0	1	1
Red Helen	*Papilio helenus*	Summer+Monsoon	R	0	0	1	0	1	1	1	0
Blue Mormon	*Papilio polymnestor*	All	VC	1	1	1	1	1	1	1	1
Common Mormon	*Papilio polytes*	All	VC	1	1	1	1	1	1	1	1
**Family: Pieridae (N = 14)**											
Common Albatross	*Appias albina*	Summer+Winter	R	0	1	1	1	1	0	0	0
Plain Pufin	*Appias indra*	Winter	VR	1	0	0	0	0	0	0	0
Striped Albatross	*Appias libythea*	Winter	R	0	0	0	1	1	0	0	0
Common Emigrant	*Catopsilia pomona*	All	VC	1	1	1	1	1	1	1	1
Mottled Emigrant	*Catopsilia pyranthe*	Winter	NC	1	1	1	1	1	0	0	0
Common Gull	*Cepora nerissa*	All	VC	1	1	1	1	1	1	1	1
Common Jezebel	*Delias eucharis*	All	C	1	1	1	1	1	1	1	1
Common Grass Yellow	*Eurema hecabe*	All	VC	1	1	1	1	1	0	1	1
Spotless Grass Yellow	*Eurema laeta*	Summer+Winter	NC	1	0	1	1	1	0	1	0
Great Orange Tip	*Hebomoia glaucippe*	All	NC	1	1	1	0	1	1	1	0
White Orange Tip	*Ixias marianne*	Summer+Winter	C	1	1	1	1	1	0	1	0
Yellow Orange Tip	*Ixias pyrene*	Summer+Winter	C	1	1	1	1	1	0	1	0
Psyche	*Leptosia nina*	All	C	1	1	1	1	1	1	1	1
Common Wanderer	*Pareronia hippia*	All	VC	1	1	1	1	1	1	1	0
**Family: Riodinidae (N = 1)**											
Double Banded Judy	*Abisara bifasciata*	Monsoon+Winter	C	1	0	1	1	1	1	0	0

**Table 4.** Activity chart for butterflies of Matheran observed during the survey. Colour codes correspond to Table 2.

**Scientific Name**	**Mud Puddling**	**Basking**	**Feeding**					
			**Nectaring**	**Tree Sap**	**Carcass**	**Animal Waste (other than that of birds)**	**Bird Droppings**	**Rotten Fruits**
**Family Hesperiidae**								
*Arnetta vindhiana*	+	+	+				+	+
*Badamia exclamationis*	+		+		+		+	
*Bibasis sena*			+					
*Burara jaina*	+		+				+	+
*Caltoris kumara*			+				+	
*Caprona ransonnetti*	+	+	+				+	+
*Celaenorrhinus ambareesa*	+	+	+				+	+
*Celaenorrhinus leucocera*		+	+					
*Celaenorrhinus ruficornis*		+	+					
*Coladenia indrani*	+	+	+				+	+
*Hasora badra*			+		+		+	
*Hasora chromus*	+		+				+	+
*Hasora vitta*			+					
*Iambrix salsala*		+	+				+	
*Matapa aria*	+		+				+	
*Pelopidas conjuncta*	+	+	+				+	
*Pelopidas mathias*	+	+	+				+	
*Sarangesa dasahara*	+	+	+				+	+
*Sarangesa purendra*	+	+	+				+	+
*Spialia galba*		+	+				+	
*Suastus gremius*			+				+	
*Tapena thwaitesi*	+	+	+		+	+	+	+
*Taractrocera ceramas*		+	+				+	
*Telicota bambusae*	+	+	+				+	
*Udaspes folus*	+	+	+		+		+	+
**Family Lycaenidae**								
*Acytolepis puspa*	+	+	+		+	+	+	+
*Amblypodia anita*	+	+	+		+	+	+	
*Anthene lycaenina*	+	+	+					
*Arhopala amantes*	+	+						+
*Arhopala centaurus*	+	+						+
*Caleta decidia*	+		+	+	+	+	+	
*Castalius rosimon*	+	+	+	+	+	+	+	
*Catochrysops strabo*	+	+	+		+	+		+
*Cheritra freja*		+	+					
*Chilades lajus*	+	+	+					
*Chliaria othona*	+	+	+					
*Curetis dentata*	+	+						+
*Curetis thetis*	+	+						+
*Deudorix epijarbas*	+	+	+				+	+
*Euchrysops cnejus*	+		+					
*Everes lacturnus*	+		+			+		
*Freyeria putli*	+	+	+					
*Iraota timoleon*	+	+	+		+	+		+
*Jamides bochus*	+		+			+	+	
*Jamides celeno*	+		+			+	+	
*Lampides boeticus*	+	+	+		+	+	+	+
*Leptotes plinius*	+	+	+			+	+	+
*Loxura atymnus*		+						+
*Luthrodes pandava*	+		+					
*Megisba malaya*	+		+			+		
*Nacaduba beroe*	+					+		
*Nacaduba kurava*	+					+		
*Petrelaea dana*	+					+		
*Prosotas dubiosa*	+		+			+		
*Prosotas nora*	+		+			+		
*Rapala iarbus*	+	+	+					
*Rapala manea*	+	+						
*Rapala varuna*	+	+			+			
*Rathinda amor*		+	+					+
*Spalgis epius*	+						+	
*Spindasis lohita*	+	+	+		+			
*Spindasis vulcanus*	+	+	+		+			
*Surendra quercetorum*	+		+		+			
*Tajuria cippus*	+	+			+	+		
*Talicada nyseus*	+	+	+					
*Tarucus ananda*	+							
*Virachola isocrates*	+	+	+		+	+	+	+
*Virachola perse*	+	+	+		+	+	+	+
*Zizeeria karsandra*	+	+	+			+		
*Zizina otis*	+	+	+			+		
*Zizula hylax*	+	+	+			+		
**Family Nymphalidae**								
*Ariadne ariadne*	+	+	+					
*Ariadne merione*	+	+	+					
*Athyma inara*	+	+						
*Athyma perius*	+	+						
*Charaxes psaphon*	+	+		+	+	+		+
*Charaxes solon*	+	+		+	+	+		+
*Cupha erymanthis*	+	+	+					
*Cyrestis thyodamas*	+	+			+			
*Danaus chrysippus*	+	+	+					
*Danaus genutia*	+	+	+					
*Euploea core*	+	+	+					
*Euploea klugii*	+	+	+					
*Euploea sylvester*	+	+	+					
*Euthalia aconthea*	+	+	+	+	+	+	+	+
*Euthalia lubentina*	+	+	+	+	+	+	+	+
*Hypolimnas bolina*	+	+	+		+	+	+	+
*Hypolimnas misippus*	+	+	+		+	+	+	+
*Junonia almana*	+	+	+					+
*Junonia atlites*	+	+	+					
*Junonia iphita*	+	+	+		+			+
*Junonia lemonias*	+	+	+					+
*Kallima horsfieldii*	+	+		+	+	+		+
*Lethe europa*					+	+		+
*Lethe rohria*					+	+		+
*Libythea myrrha*	+	+						
*Melanitis leda*					+	+		+
*Moduza procris*	+	+	+		+	+	+	+
*Mycalesis mineus*	+	+	+		+		+	+
*Mycalesis perseus*	+	+	+		+		+	+
*Mycalesis visala*	+	+	+		+		+	+
*Neptis hylas*	+	+	+		+			+
*Neptis jumbah*	+	+	+		+			+
*Parantica aglea*	+	+	+					
*Phaedyma columella*	+	+	+		+			+
*Phalanta phalantha*	+	+	+		+	+		+
*Polyura bharata*	+	+		+	+	+		+
*Rohana parisatis*	+	+				+		+
*Symphaedra nais*	+	+	+		+		+	+
*Tanaecia lepidea*	+	+		+	+	+	+	+
*Tirumala limniace*	+	+	+					
*Tirumala septentrionis*	+	+	+					
*Vanessa cardui*	+	+	+					+
*Ypthima baldus*		+	+					+
*Ypthima huebneri*		+	+					+
**Family Papilionidae**								
*Graphium agamemnon*	+	+	+		+	+		
*Graphium doson*	+		+		+	+		
*Graphium teredon*	+		+		+	+		
*Pachliopta aristolochiae*		+	+					
*Pachliopta hector*		+	+					
*Papilio clytia*	+		+					
*Papilio demoleus*	+	+	+					
*Papilio helenus*	+	+	+					
*Papilio polymnestor*	+	+	+					
*Papilio polytes*	+	+	+					
**Family Pieridae**								
*Appias albina*	+	+	+					
*Appias indra*	+	+	+					
*Appias libythea*	+	+	+					
*Catopsilia pomona*	+		+					
*Catopsilia pyranthe*	+		+					
*Cepora nerissa*	+	+	+					
*Delias eucharis*		+	+					
*Eurema hecabe*			+			+	+	
*Eurema laeta*			+			+	+	
*Hebomoia glaucippe*	+	+	+					
*Ixias marianne*		+	+					
*Ixias pyrene*		+	+					
*Leptosia nina*			+					
*Pareronia hippia*	+	+	+					
**Family Riodinidae**								
*Abisara bifasciata*	+	+						

**Table 5.** List of scheduled species under the Wildlife (Protection) Act, 1972, India.

**S r. No.**	**Common Name**	**Scientific Name**	**Schedule (Part)**
1	Orange-tailed awlet	*Bibasis sena*	2 (2)
2	Plain Banded Awl	*Hasora vitta*	4
3	Striped Albatross	*Appias libythea*	4
4	Plain Puffin	*Appias indra*	2 (2)
5	Crimson Rose	*Pachliopta hector*	1 (4)
6	Long Banded Silverline	*Spindasis lohita*	2 (2)
7	Dark Pierrot	*Tarucus ananda*	4
8	Gram Blue	*Euchrysops cnejus*	2 (2)
9	Lime blue	*Chilades lajus*	2
10	Peacock Royal	*Tajuria cippus*	2 (2)
11	Orchid Tit	*Chliaria othona*	1 (4)
12	Indigo Flash	*Rapala varuna*	2 (2)
13	Gaudy Baron	*Euthalia lubentina*	4
14	Grey Count	*Tanaecia lepidea*	2 (2)
15	Danaid Eggfly	*Hypolimnas misippus*	1

**Table 6.** List of the butterfly species of Matheran common between Smith (1882), Betham (1894), Padhye et al. (2013) and the current study.

**Accepted Name**	**Smith (1882)**	**Betham (1894)**	**Padhye et al. (2013)**	**Our list**	**Remarks**
*Abisara echerius*	−	*Abisara suffusa*	−	−	
*Acytolepis puspa*	−	*Cyaniris puspa*	−	*Acytolepis puspa*	
*Anthene lycaenina*	−	−	*Anthene lycaenina*	*Anthene lycaenina*	
*Appias albina*	*Huphina albina*	−	−	*Appias albina*	A doubtful generic allocation by Smith (1882)
*Appias paulina*	*Catophaga paulina*	−	−	−	
*Ariadne ariadne*	*Ergolis ariadne*	*Ergolis ariadne*	−	*Ariadne ariadne*	
*Ariadne merione*	−	−	*Ariadne merione*	*Ariadne merione*	
*Athyma perius*	*Athyma perius*	*Athyma perius*	−	*Athyma perius*	
*Badamia exclamationis*	−	*Badamia exclamationis*	−	*Badamia exclamationis*	
*Belenois aurota*	*Belenois mesentina*	−	−	−	
*Bibasis sena*	−	*Bibasis sena*	−	*Bibasis sena*	
*Byblia ilithyia*	*Byblia ilithyia*	−	−	−	
*Caleta roxus*	*Castalius roxus*	−	−	−	
*Castalius rosimon*	*Castalius rosimon*	*Castalius rosimon*	−	*Castalius rosimon*	
*Catopsilia pomona*	*Catopsilia hilaria*	*Catopsilia catilia*	*Catopsilia pomona*	*Catopsilia pomona*	
*Catopsilia pyranthe*	*Catopsilia phillipina*	−	*Catopsilia pyranthe*	*Catopsilia pyranthe*	
*Celaenorrhinus ambareesa*	−	*Celenorrhinus ambareesa*	−	*Celaenorrhinus ambareesa*	
*Cepora nerissa*	*Huphina phryne*	*Huphina phryne*	−	*Cepora nerissa*	
*Charaxes psaphon*	−	*Charaxes imna*	−	*Charaxes psaphon*	
*Cyrestis thyodamas*	* Cyrestis *	−	−	*Cyrestis thyodamas*	Smith (1882) mentions only generic name. Possibly *Cyrestis thyodamas*
*Danaus chrysippus*	*Danais chrysippus*	*Danais chrysippus*	−	*Danaus chryssipus*	Erroneous generic name by Smith (1882) and Betham (1894)
*Danaus genutia*	*Danais genutia*	*Danais genutia*	*Danaus genutia*	*Danaus genutia*	Erroneous generic name by Smith (1882) and Betham (1894)
*Delias eucharis*	−	*Delias eucharis*	−	*Delias eucharis*	
*Deudorix epijarbas*	−	*Deudorix epijarbas*	−	*Deudorix epijarbas*	
*Euchrysops cnejus*	*Catochrysops cnejus*	*Catochrysops cnejus*	−	*Euchrysops cnejus*	
*Euploea core*	−	*Euploea core*	−	*Euploea core*	
*Eurema brigitta*	−	−	*Eurema brigitta*	−	
*Eurema hecabe*	*Terias hecabe*	−	*Eurema hecabe*	*Eurema hecabe*	
*Graphium agamemnon*	*Papilio agamemnon*	−	*Graphium agamemnon*	*Graphium agamemnon*	
*Graphium teredon*	*Papilio sarpedon*	−	*Graphium sarpedon*	*Graphium teredon*	
*Hasora chromus*	−	*Parata chromus*	−	*Hasora chromus*	
*Hebomoia glaucippe*	*Hebomia glaucippe*	−	−	*Hebomoia glaucippe*	Erroneous generic name by Smith (1882)
*Hypolimnas bolina*	−	*Hypolimnas bolina*	*Hypolimnas bolina*	*Hypolimnas bolina*	
*Hypolimnas misippus*	*Hypolimnas misippus*	*Hypolimnas misippus*	*Hypolimnas misippus*	*Hypolimnas misippus*	
*Iraota timoleon*	*Iraota mecenas*	−	−	*Iraota timoleon*	
*Jamides bochus*	−	−	*Jamides bochus*	*Jamides bochus*	
*Jamides celeno*	−	−	*Jamides celeno*	*Jamides celeno*	
*Junonia almana*	−	*Junonia almana, v. asterie*	*Junonia almana*	*Junonia almana*	
*Junonia iphita*	*Precis iphita*	−	*Junonia iphita*	*Junonia iphita*	
*Junonia lemonias*	*Junonia lemonias*	*Junonia lemonias*	*Junonia lemonias*	*Junonia lemonias*	
*Junonia oenone*	*Junonia oenone*	*Junonia oenone*	−	−	
*Junonia orithyia*	*Junonia orithyia*	−	−	−	
*Kallima horsfieldii*	*Kallima horsefieldii*	*Kallima horsefieldii*	−	*Kallima horsfieldii*	Erroneous specific name in Smith (1882) and Betham (1894)
*Leptosia nina*	−	*Leptosia xiphia*	−	*Leptosia nina*	
*Leptotes plinius*	*Tarucus plinius*	*Tarucus plinius*	−	*Leptotes plinius*	
*Lethe rohria*	−	*Lethe nilgheriensis*	−	*Lethe rohria*	
*Luthrodes pandava*	−	−	*Chilades pandava*	*Luthrodes pandava*	
*Matapa aria*	*Matapa aria*	−	−	*Matapa aria*	
*Melanitis leda*	*Melanitis leda*	−	*Melanitis leda*	*Melanitis leda*	
*Melanitis leda*	*Melanitis ismene*	*Melanitis ismene*	−	*Melanitis leda*	
*Mycalesis mineus*	*Mycalesis mineus*	−	−	*Mycalesis mineus*	
*Mycalesis perseus*	−	*Mycalesis perseus*	−	*Mycalesis perseus*	
*Neptis hylas*	*Neptis varmona*	*Neptis varmona, v. eurymene*	*Neptis hylas*	*Neptis hylas*	
*Neptis jumbah*	−	*Neptis jumbah*	−	*Neptis jumbah*	
*Pachliopta aristolochiae*	−	−	*Pachiliopta aristolochae*	*Pachliopta aristolochiae*	Erroneous generic and specific name in Padhye et al. (2013)
*Pachliopta hector*	*Papilio hector*	−	*Pachliopta hector*	*Pachliopta hector*	
*Papilio ambrax*	*Papilio epius*	−	−	−	
Papilio clytia form dissimilis	Papilio form dissimilis	−	−	Papilio clytia form dissimilis	
Papilio clytia form clytia	Papilio form panope	−	Papilio clytia form clytia	Papilio clytia form clytia	
*Papilio deiphobus*	*Papilio deiophobus*	−	−	−	This could be misidentification as the species is distributed in the Philippines, Moluccas and some parts of West Papua.
*Papilio demoleus*	−	−	*Papilio demoleus*	*Papilio demoleus*	
*Papilio iswara*	*Papilio iswara*	−	−	−	This could be misidentification as the species is distributed over the Sundaland.
*Papilio polymnestor*	*Papilio polymnestor*	*Papilio polymnestor*	*Papilio polymnestor*	*Papilio polymnestor*	
*Papilio polytes*	*Papilio pammon*	*Papilio Polytes*	*Papilio Polytes*	*Papilio polytes*	
*Parantica aglea*	*Danais aglea*	*Danais melanoides*	*Parantica aglea*	*Parantica aglea*	
*Pareronia valeria*	*Eronia valeria*	−	−	−	
*Pelopidas agna*	*Chapra agna*	−	−	−	
*Pelopidas mathias*	−	*Chapra mathias*	−	*Pelopidas mathias*	
*Phaedyma columella*	−	*Neptis ophiana*	−	*Phaedyma columella*	
*Phalanta phalantha*	*Atella phalanta*	*Atella phalantha*	−	*Phalanta phalantha*	Erroneous specific name by Smith (1882)
*Polyura bharata*	*Charaxes athamas*	−	−	*Polyura bharata*	
*Prosotas nora*	−	−	*Prosotas nora*	*Prosotas nora*	
*Sarangesa purendra*	*Sarangesa purendra*	*Sarangesa purendra*	−	*Sarangesa purendra*	
*Spialia galba*	*Hesperia galba*	−	−	*Spialia galba*	
*Spindasis lohita*	*Aphneus lohita*	−	−	*Spindasis lohita*	
*Tarucus theophrastus*	*Tarucus theophrastus*	−	−	−	
*Tirumala limniace*	*Danais limniace*	*Danais limniace*	*Tirumala limniace*	*Tirumala limniace*	
*Udaspes folus*	*Udaspes folus*	*Udaspes folus*	−	*Udaspes folus*	
*Vanessa indica*	*Pyrameis indica*	−	−	−	
*Ypthima philomela*	*Ypthima philomela*	*Ypthima philomela*	−	−	
*Ypthima singala*	*Ypthima singala*	−	−	−	
*Zeltus amasa*	*Zeltus etolus*	−	−	−	
*Not found*	*Danais careta*	−	−	−	Doubtful record by Smith (1882). Put ? by Betham (1894)
*Not found*	* Poritia *	−	−	−	
*Not found*	*Lampides elianus*	−	−	−	
*Not found*	−	*Terias esiope*	−	−	
*Not found*	−	*Isoteinon nilgheriensis*	−	−	Monotypic genus contains *Isoteinon lamprospilus*
