# Supplementary material for: Finding the forgotten gems: revisiting the butterflies of Matheran after 125 years with introduction to novel colour barcode for depicting seasons and activity of the Indian butterflies
Source: Biodivers Data J. 2020 Aug 7;8:e54333. doi: 10.3897/BDJ.8.e54333 (PMC7431446; doi:10.3897/BDJ.8.e54333)
Supplement: Supplementary material 1 — Percentage matrix fill and percentage contributions from the SDR-simplex analyses of family-wise and overall species richness. [file bdj-08-e54333-s001.docx]

Supplementary 2: Percentage matrix fill and percentage contributions from the SDR-simplex analyses of family-wise and overall species richness.

|  | Matrix fill (%) | Similarity (%) | Relativised Species replacement | Relativised richness difference | Relativised beta diversity | Relativised richness agreement | Relativised nestedness | Percentage anti-nestedness fraction | Relativised strict nestedness | Percentage richness identity fraction |
| --- | --- | --- | --- | --- | --- | --- | --- | --- | --- | --- |
| Hesperiidae | 74.00 | 65.91 | 14.33 | 19.76 | 34.10 | 80.24 | 85.67 | 0.00 | 85.67 | 0.00 |
| Lycaenidae | 59.24 | 49.10 | 18.91 | 31.99 | 50.90 | 68.01 | 81.09 | 0.00 | 79.17 | 1.92 |
| Nymphalidae | 75.00 | 70.58 | 14.78 | 14.64 | 29.42 | 85.36 | 85.22 | 0.00 | 78.22 | 7.00 |
| Papilionidae | 72.50 | 59.54 | 14.63 | 25.83 | 40.46 | 74.17 | 85.37 | 0.00 | 77.28 | 8.09 |
| Pieridae | 72.32 | 65.61 | 6.44 | 27.95 | 34.39 | 72.05 | 93.56 | 0.00 | 84.96 | 8.59 |
| Overall | 69.11 | 61.67 | 20.12 | 18.21 | 38.33 | 81.79 | 79.88 | 0.00 | 79.88 | 0.00 |
