# Supplementary material for: Finding the forgotten gems: revisiting the butterflies of Matheran after 125 years with introduction to novel colour barcode for depicting seasons and activity of the Indian butterflies
Source: Biodivers Data J. 2020 Aug 7;8:e54333. doi: 10.3897/BDJ.8.e54333 (PMC7431446; doi:10.3897/BDJ.8.e54333)
Supplement: Supplementary material 2 — Similarity-Richness difference-Species replacement simplex plot for overall butterfly diversity of Matheran showing high similarity. Points denote pair of sites (N = 28) [file bdj-08-e54333-s002.docx]

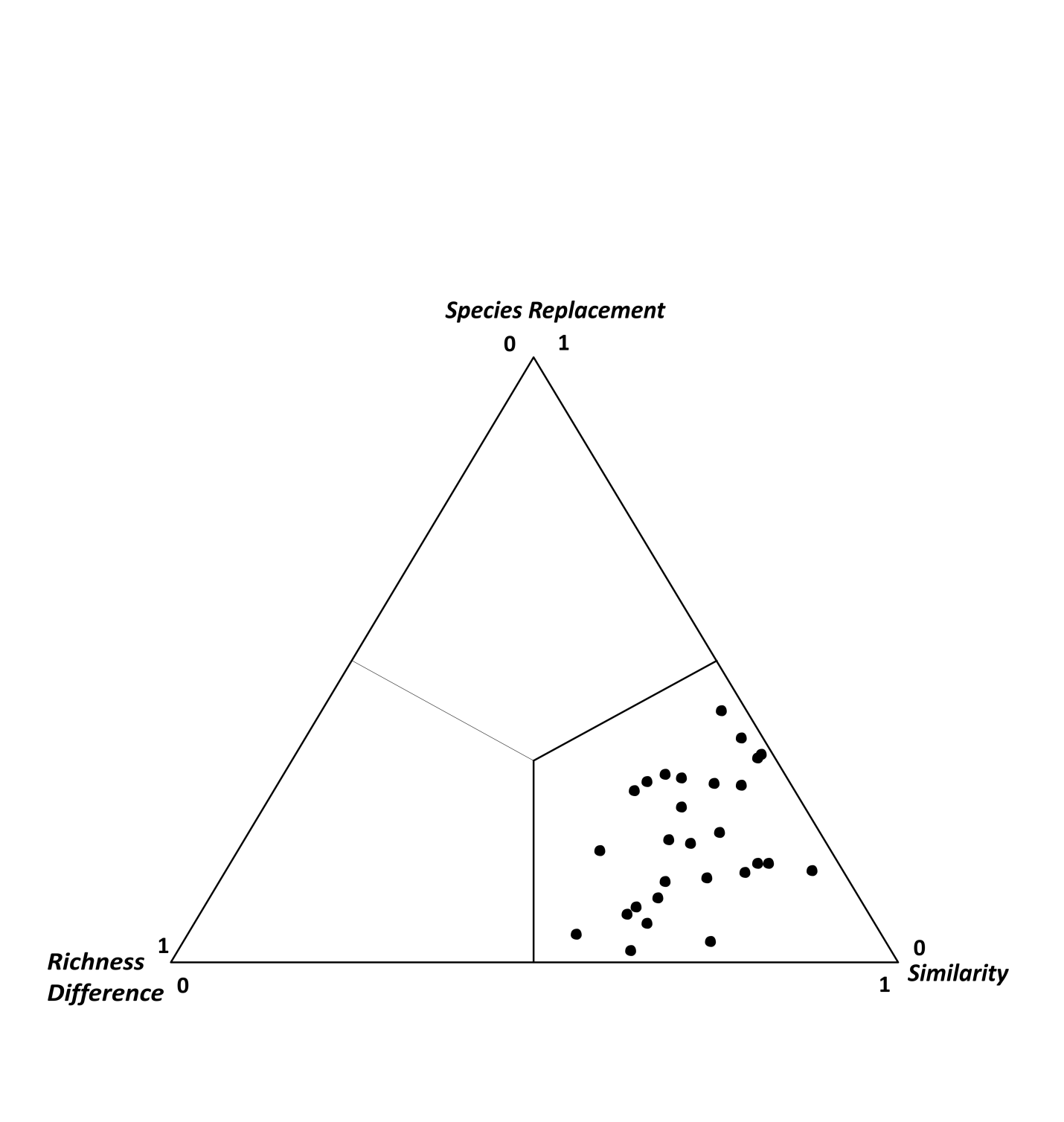


Supplementary 3: Similarity-Richness difference-Species replacement simplex plot for overall butterfly diversity of Matheran showing high similarity. Points denote pair of sites (N = 28)
